# Sex differences based on the timing of invasive management among patients with non-ST-elevation acute coronary syndrome: an individual patient data meta-analysis

**DOI:** 10.1093/ehjopen/oeaf059

**Published:** 2025-05-17

**Authors:** Graziella Pompei, Gregory B Mills, Christos P Kotanidis, Shamir Mehta, Denise Tiong, Erik A Badings, Thomas Engstrøm, Arnoud W J van‘t Hof, Dan Høfsten, Lene Holmvang, Alexander Jobs, Lars Køber, Dejan Milasinovic, Aleksandra Milosevic, Goran Stankovic, Holger Thiele, Roxana Mehran, Vijay Kunadian

**Affiliations:** Translational and Clinical Research Institute, Faculty of Medical Sciences, Newcastle University, Newcastle upon Tyne NE2 4HH, UK; Cardiovascular Institute, Azienda Ospedaliero-Universitaria di Ferrara, Via Aldo Moro 8, 44124 Cona, Ferrara, Italy; Cardiology Unit, Ospedale Santa Maria Delle Croci, Viale Vincenzo Randi 5, 48121 Ravenna, Italy; Translational and Clinical Research Institute, Faculty of Medical Sciences, Newcastle University, Newcastle upon Tyne NE2 4HH, UK; Cardiothoracic Centre, Freeman Hospital, Newcastle upon Tyne Hospitals NHS Foundation Trust, Freeman Rd, High Heaton, Newcastle upon Tyne NE7 7DN, UK; Translational and Clinical Research Institute, Faculty of Medical Sciences, Newcastle University, Newcastle upon Tyne NE2 4HH, UK; Cardiothoracic Centre, Freeman Hospital, Newcastle upon Tyne Hospitals NHS Foundation Trust, Freeman Rd, High Heaton, Newcastle upon Tyne NE7 7DN, UK; Population Health Research Institute, McMaster University and Hamilton Health Sciences, 237 Barton Street, East Hamilton, ON, L8L 2X2, Canada; Population Health Research Institute, McMaster University and Hamilton Health Sciences, 237 Barton Street, East Hamilton, ON, L8L 2X2, Canada; Department of Cardiology Research, Deventer Hospital, Nico Bolkesteinlaan 75, 7416 SE Deventer, The Netherlands; Department of Cardiology, Rigshospitalet, Copenhagen University Hospital, Blegdamsvej 9, 2100 Copenhagen, Denmark; Department of Cardiology, Maastricht University Medical Center, P. Debyelaan 25, 6229 ET Maastricht, The Netherlands; Department of Cardiology, Zuyderland Medical Center, Henri Dunantstraat 5, 6419 PC Heerlen, The Netherlands; Cardiovascular Research Institute Maastricht, Universiteitssingel 50, 6229 ER Maastricht, The Netherlands; Department of Cardiology, Rigshospitalet, Copenhagen University Hospital, Blegdamsvej 9, 2100 Copenhagen, Denmark; Department of Cardiology, Rigshospitalet, Copenhagen University Hospital, Blegdamsvej 9, 2100 Copenhagen, Denmark; Faculty of Health and Medical Sciences, University of Copenhagen, Blegdamsvej 3B, 2200 Copenhagen, Denmark; Heart Center Leipzig at Leipzig University and Leipzig Heart Science, Strümpellstr. 39, 04289 Leipzig, Germany; Department of Cardiology, Rigshospitalet, Copenhagen University Hospital, Blegdamsvej 9, 2100 Copenhagen, Denmark; Faculty of Medicine, University of Belgrade, dr Subotića starijeg 8, 11000 Belgrade, Serbia; Department of Cardiology, Clinical Center of Serbia, 26 Visegradska, 11000 Belgrade, Serbia; Faculty of Medicine, University of Belgrade, dr Subotića starijeg 8, 11000 Belgrade, Serbia; Department of Cardiology, Clinical Center of Serbia, 26 Visegradska, 11000 Belgrade, Serbia; Faculty of Medicine, University of Belgrade, dr Subotića starijeg 8, 11000 Belgrade, Serbia; Department of Cardiology, Clinical Center of Serbia, 26 Visegradska, 11000 Belgrade, Serbia; Heart Center Leipzig at Leipzig University and Leipzig Heart Science, Strümpellstr. 39, 04289 Leipzig, Germany; Mount Sinai School of Medicine, One Gustave L. Levy Place, Box 1030, New York, NY 10029-6574, USA; Translational and Clinical Research Institute, Faculty of Medical Sciences, Newcastle University, Newcastle upon Tyne NE2 4HH, UK; Cardiothoracic Centre, Freeman Hospital, Newcastle upon Tyne Hospitals NHS Foundation Trust, Freeman Rd, High Heaton, Newcastle upon Tyne NE7 7DN, UK

**Keywords:** Non-ST-elevation acute coronary syndrome, Sex, Female, Early invasive strategy, Delayed invasive strategy, Percutaneous coronary intervention

## Abstract

**Aims:**

Studies investigating the timing of coronary angiography in non-ST-elevation acute coronary syndrome (NSTE-ACS) have not evaluated sex differences. This study aims to investigate the sex-related differences in outcomes of NSTE-ACS patients undergoing early or delayed invasive management.

**Methods and results:**

An individual patient data (IPD) meta-analysis was performed after systematic review of randomized controlled trials (RCTs) comparing early vs. delayed invasive strategy among NSTE-ACS patients. The primary endpoint was a composite of all-cause death or myocardial infarction (MI) at 6 months. Secondary endpoints included all-cause death, MI, recurrent ischaemia, stroke, and major bleeding. One-stage, random-effects Cox models were conducted. This meta-analysis was registered with PROSPERO (CRD42023468604). Six RCTs including 6654 patients were identified, of whom 2257 (33.9%) were females with a median age of 69 years [interquartile range (IQR) 60–76], significantly higher than males (64.5 years, IQR 55–72.1, *P* < 0.001). Among patients undergoing early strategy, there was no sex difference in the occurrence of the primary [Hazard ratio (HR) 1.08, 95% confidence interval (CI) 0.83–1.41, *P* = 0.560] and secondary endpoints. Among patients undergoing delayed strategy, there was no difference in the occurrence of the primary endpoint (HR 1.12, 95% CI 0.88–1.43, *P* = 0.350). Female sex undergoing delayed strategy was associated with higher risk of recurrent ischaemia (HR 1.52, 95% CI 1.06–2.19, *P* = 0.023) and major bleeding (HR 1.88, 95% CI 1.22–2.87, *P* = 0.004) using univariable analysis but not using multivariable analysis.

**Conclusion:**

No sex-related differences in the composite of all-cause death or MI were identified among NSTE-ACS patients undergoing early and delayed invasive management.

## Graphical Abstract

**Graphical Abstract:**
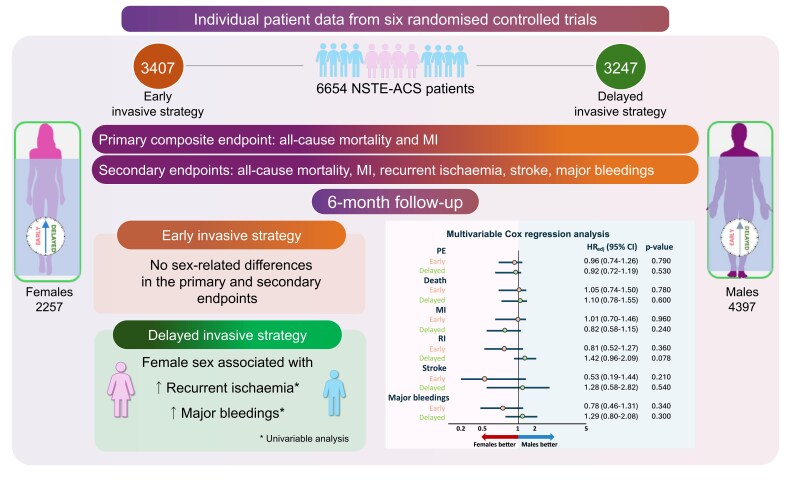

## Introduction

Ischaemic heart disease is the leading cause of mortality in females globally.^1^ Females presenting with myocardial infarction (MI) have higher risk of major adverse events compared with males.^2,3^ Females with non-ST-elevation acute coronary syndrome (NSTE-ACS) are older than males, and have a higher burden of comorbidities and complications following invasive strategy.^4–7^ Despite international guidelines recommending equality in the management of ACS regardless of sex,^8^ females are under-represented in studies investigating different interventional strategies and are less likely to receive evidence-based therapies.^4,8–11^

The use of routine invasive coronary angiography for high-risk NSTE-ACS, in comparison to a selective or ‘ischaemia-guided’ strategy, is associated with reductions in ischaemic outcomes.^12–14^ Current guidelines recommend (Class IIa) an early invasive strategy (within 24 h) in the presence of any high-risk criteria, such as diagnosis of non-ST-elevation myocardial infarction (NSTEMI) based on high-sensitivity cardiac troponin, dynamic ST-segment/T waves changes, transient ST-elevation, and Global Registry of Acute Coronary Events (GRACE) risk score >140.^8^ Over the past decade, several studies have reinforced the recommendation that complete coronary revascularization should be the preferred approach over culprit-only revascularization in ACS patients with multivessel disease. This strategy, irrespective of the timing of intervention, is aimed at reducing the risk of cardiovascular mortality, recurrent MI, and ischaemia-driven revascularization.^15,16^ We recently showed that an early invasive management in females with NSTE-ACS, compared with a delayed invasive management, was not associated with a significant reduction in the hazard for all-cause mortality or MI.^17^ In pre-specified subgroup analysis, high-risk females as assessed with GRACE score >140 or elevated cardiac biomarkers experienced significant reductions in all-cause mortality or MI at 6 months following early invasive management.^17^ However, the sex-related differences in adverse clinical outcomes between females and males with NSTE-ACS undergoing early or delayed invasive management is not known.^18–23^ This individual patient data (IPD) meta-analysis aims to investigate the sex-related differences in adverse clinical outcomes among patients undergoing early or delayed invasive strategy for the management of their NSTE-ACS.

## Methods

### Search strategy and selection criteria

The present study consists of a *post-hoc* analysis using IPD of randomized controlled trials (RCTs) enrolling NSTE-ACS patients randomly allocated to early or delayed invasive management, with a specific focus on sex-related differences. The inclusion criteria were: (1) patients with a diagnosis of unstable angina or NSTEMI (according to international guidelines in place at the time of each trial) hospitalized within 24 h after ischaemic symptoms. Diagnostic criteria included (i) electrocardiographic changes compatible with ischaemia [new ST-segment depression >1 millivolt (mV), transient ST-segment elevation or T waves inversion] and/or (ii) elevated biomarkers [troponin T > 0.05 ng/mL, troponin I greater than the upper limit of normal, myoglobin >150 µg/L, creatine kinase muscle brain (CK-MB) fraction >6% of total CK]; (2) comparison between early vs. delayed invasive strategy; (iii) random allocation; (iv) at least 30-day follow-up; and (v) minimum of 200 participants and/or 50 females. Randomized controlled trials were selected by searching MEDLINE, Web of Science and Scopus without language and date restriction. Detailed search algorithm is provided in the Supplementary material online, *Supplementary methods*.

Studies were screened for eligibility by two independent reviewers (GBM and VK). After systematic evaluation of the inclusion and exclusion criteria, the principal investigators of all eligible studies were invited to share published and/or unpublished IPD. Variables of interest were selected at the study protocol stage according to clinical relevance and consistency across trials. Data accuracy was assessed for completeness and integrity before being merged into a single database. This IPD meta-analysis was conducted according to the Preferred Reporting Items of Systematic Reviews and Meta-analyses (PRISMA) statement (see Supplementary material online, *Table S1*). The protocol was registered on the International Prospective Register of Systematic Reviews (PROSPERO, CRD42023468604).

### Comparison groups and outcomes

Early and delayed invasive strategies were defined according to the original definitions of each randomized controlled trial (RCT) and based on the time interval between randomization and coronary angiography. In case of studies randomizing patients to three different arms, patients allocated to the immediate invasive strategy arm were combined with those undergoing an early invasive strategy.

All patients were prescribed optimal medical therapy regardless of the revascularization strategy. A median follow-up was used according to the follow-up length reported in each RCT. The primary endpoint was a composite of all-cause death or MI. The secondary endpoints included individual clinical outcomes such as all-cause death, MI, stroke, recurrent ischaemia, and major bleeding events. Clinical endpoints were as defined originally by the included trials (*Table 1*). Overall, across the RCTs recurrent ischaemia was defined as recurrent chest pain despite optimal medical therapy associated with or without electrocardiogram (ECG) changes and requiring repeat hospitalization or additional intervention. The definition is heterogeneous across the studies, but it did not coincide with those used to define MI.

**Table 1:** Endpoint definitions in the included trials

Study	Patients	Invasive strategy timing	Primary endpoint	Secondary endpoints	Non-fatal MI	Recurrent ischaemia	Bleeding
ELISA Van’t Hof *et al.*^19^	*n* = 220 Early group: *n* = 109 Delayed group: *n* = 111	**Early group:** Angiography within 12 h (median 6 h) **Late group:** Angiography after 24–48 h (median 50 h)	Enzymatic infarct size	Death and recurrent MI at 30 days	**Spontaneous recurrent MI:** A new CK-MB rise >6% of total CK, whenever CK was > 200 U/L (men) or >170 U/L (women) **MI after PCI:** A new rise in CK-MB >3 times the above mentioned 6% level **MI after CABG:** New Q-waves on the ECG	Requirement for repeat angiography and subsequent repeat TLR	**Major bleeding:** Need for at least 2 U of blood and a fall in Hb of >2 mmol/L, corrective groin surgery, gastro-intestinal or retroperitoneal bleeding
TIMACS Mehta *et al.*^23^	*n* = 3031 Early group: *n* = 1593 Delayed group: *n* = 1438	**Routine early group:** Angiography ≤24 h (median 14 h) **Delayed group:** Angiography≥36 h (median 50 h)	First occurrence of composite of death, new MI, or stroke at 6 months	First occurrence of composite of death, MI, or refractory ischaemia and the composite of death, MI, stroke, refractory ischaemia, or repeat intervention at 6 months Each of the individual components analysed separately	**MI within 24 h:** New ischaemic symptoms >20 min and new/recurrent ST ↑/↓ > 0.1 mV in >2 contiguous leads **MI between 24 h and 7 days:** Ischaemic symptoms >20 min and CK-MB > 2 × ULN or >50% above the previous lowest level in patients with already ↑ biomarkers or new/recurrent ST ↑/↓ > 0.1 mV or new Q-waves in >2 contiguous leads **MI in patients with no biomarkers ↑ at baseline: ↑** biomarkers (Tn, CK-MB, CK) > 2 times the ULN with at least 1 of the following: ischaemic symptoms, pathological Q-waves, ECG changes indicative of ischaemia, coronary intervention or pathological findings of an acute MI (this latter definition was also used for new MI occurring 7 days after randomization) **MI after PCI:** CK-MB >3× ULN or ↑ by 50% from the pre-procedural lowest level and >3× ULN in patients with already ↑ enzymes or new ST ↑ or Q-waves in >2 contiguous leads **MI after CABG:** CK-MB >5× ULN or ↑ by 50% from the pre-procedural lowest level and >5× ULN in patients with already ↑ enzymes or new Q-wave in >2 contiguous leads	**Refractory ischaemia:** Recurrent symptoms >5 min, while on OMT (at least 2 medications), with ECG changes and requiring an additional intervention (thrombolytic therapy, cardiac catheterization, IABP insertion, or revascularisation) within 48 h of the onset of this episode	**Major bleeding:** clinically overt bleeding with at least one of the following: Fatal bleed Symptomatic intracranial bleed Retroperitoneal bleed Intraocular bleed leading to significant vision loss Decrease in Hb ≥ 3.0 g/dL Bleed requiring transfusion of 2 or more RBCs units or equivalent of whole blood Bleeding requiring surgical intervention
LIPSIA-NSTEMI Thiele *et al.*^21^	*n* = 600 Immediate group: *n* = 200 Early group: *n* = 200 Selective group: *n* = 200	**Immediate group:** Angiography<2 h (median 1.1 h) **Early group:** Angiography 10–48h (median 18.3h) **Selective invasive group:** Patients initially treated medically (median 67.2 h)	CK-MB activity during index hospitalization for each patient. In addition, the infarct size was estimated based on the AUC of CK-MB release	Composite of (i) death and non-fatal MI; (ii) death, non-fatal MI, and refractory ischaemia; and (iii) death, non-fatal MI, refractory ischaemia, and rehospitalization for UA within 6 months	**Non-fatal MI:** Any recurrent major myocardial necrosis occurring either spontaneously or in the setting of revascularization. Two definitions were used: **In-hospital re-MI:** Occurrence of any of the following: new Q-waves in ≥2 contiguous leads plus ischaemic symptoms >20 min new ST ↑ in ≥2 contiguous leads plus symptoms >20 min; or ↑ CK-MB >5 ULN. In patients with CK-MB >5 ULN at randomization an increase >50% was required **Re-MI after discharge:** Any new ischaemic symptoms leading to re-admission plus ↑ Tn > the 99th percentile ULN	**Refractory ischaemia:** Recurrent ischaemic symptoms >10 min despite OMT and indication for urgent invasive angiography **Rehospitalization for UA:** Any unplanned rehospitalization for UA	**Safety endpoint:** In-hospital severe/life-threatening or moderate bleeding occurring either spontaneously, PCI-related, or CABG-related as assessed by the GUSTO definition
ELISA-3	*n* = 542	**Immediate group:** Angiography <12 h (median 16.7 h)	Combined incidence of all-cause death, reinfarction and/or recurrent ischaemia at 30 days	Enzymatic infarct size assessed by cardiac Tn T (72–96 h after admission or at discharge) per cent of patients without a rise in CK-MB during admission	**Reinfarction: ↑** CK-MB on admission	Recurrent chest pain associated with new/recurrent ECG changes requiring urgent or repeat angiography or repeat hospitalization	**Major bleeding:** Bleeding with Hb drop of ≥2 mmol/L or a blood transfusion of 2 or more units
					**Early reinfarction in patients with CK-MB > ULN: ↓** CK-MB ≥50% of ULN from a prior peak level to the lowest followed by ↑ with a value greater than the sum of the preceding lowest level and three times the ULN or new Q-waves in ≥2 contiguous leads		
Badings *et al.*^18^	Immediate group: *n* = 269						
	Delayed group: *n* = 265	**Delayed group:** Angiography >48 h (median 42.7 h)					
					**Early reinfarction in patients with CK-MB ≤ ULN:** Peak CK-MB >3 times the ULN except for cases where the CK-MB release curve was unequivocally related to the chest pain episode before randomization or new Q-waves in ≥2 contiguous leads		
				Bleeding complications			
					**Late reinfarction in patients with CK-MB returned (or remained) to normal:** Peak CK-MB >3 times ULN or new Q-waves in ≥2 contiguous leads **MI in patients who underwent CABG:** New Q-waves in ≥2 contiguous leads		

RIDDLE-NSTEMI	*n* = 323	**Immediate-intervention group:** Angiography within 2 h (median 1.4 h)	Composite of all-cause death or new MI at 30 days	Combined incidence of death, new MI, and/or recurrent ischaemia at 30 days and 1 year, as well as death or new MI at 1 year	**Early new MI within 24h:** New onset of symptoms >20 min and new/recurrent ST ↑/↓ > 0.1 mV in ≥2 contiguous leads	**Recurrent ischaemia:** Repeated episodes of ischaemic symptoms >5 min if all the following applied: Patient on OMT ECG changes indicative of ongoing ischaemia Required invasive intervention	**Major bleedings:** As per TIMI bleeding classification
Milosevic *et al.*^20^	Immediate-intervention group: *n* = 162						
					**Late new MI (from 24 h to 7 days):** New onset of symptoms >20 min and ↑ Tn >20% if the initially elevated values were stable or decreasing and/or new/recurrent ST ↑/↓ > 0.1 mV or new Q-waves in ≥2 contiguous leads		
	Delayed intervention group: *n* = 161	**Delayed intervention group:** Angiography within 72 h (median 61 h)					
				Individual components of the primary and secondary endpoints and major bleedings at 30 days and 1 year	**New MI after PCI:** New ST ↑ or Q-waves in ≥2 contiguous leads and/or an ↑ Tn >20% if the initially elevated values were stable or decreasing		
					**New MI in patients whose Tn had returned to normal:** At least 1 Tn value > ULN plus symptoms, ECG changes, evidence of abnormal wall motion, and/or IC thrombus		
VERDICT	*n* = 2147	**Very early group:** Angiography within 12 h (median 4.7 h)	Combination of all-cause death, non-fatal recurrent MI, hospital admission for refractory myocardial ischaemia or HF	Invasive procedure complications during index hospitalisation (death, bleeding by the BARC criteria, non-fatal acute MI, stroke, or TIA) in addition to the occurrence of each of the following at any time: death, non-fatal acute MI, admission for refractory myocardial ischaemia, repeat coronary revascularization, or admission for HF	Occurrence of any of the following 3: ↑ and/or ↓ of cardiac biomarkers with at least 1 value >99th percentile ULN (if ↑ biomarkers at baseline, a further ↑ ≥20% is required and the peak value must exceed the previously stated limit), plus the evidence of ischaemia from at least 1 of the following: Symptoms of ischaemia ECG changes in two contiguous leads New Q-waves in two contiguous ECG leads Evidence of a new loss of viable myocardium or wall motion abnormality Sudden/unexpected cardiac death, involving cardiac arrest, often with symptoms suggestive of myocardial ischaemia, but occurring before blood samples could be obtained, or at a time before the appearance of biomarkers in the blood and accompanied by: Presumably new ST ↑ New LBBB Evidence of fresh thrombus Pathological findings of a MI	**Hospital admission for refractory myocardial ischaemia:** Hospitalisation due to chest pain not associated with MI and a clinical suspicion resulting in coronary angiography	
Kofoed *et al.*^22^	Very early group: *n* = 1075						
	Standard group: *n* = 1072	**Standard care group:** Angiography within 48–72 h (median 61.6 h)					
						**Repeat coronary revascularisation:** Non-acute PCI or CABG, excluding revascularisation planned during index hospitalisation or in preparation for treatment of valvular dysfunction	

Abbreviations. MI, myocardial infarction; CK-MB, creatine kinase muscle/brain; PCI, percutaneous coronary intervention; ULN, upper limit of normal; Hb, haemoglobin; RBCs, red blood cells; TIA, transient ischaemic attack; LBBB, left bundle branch block; UA, unstable angina; Tn, troponin; IC, intracoronary; BARC, Bleeding Academic Research Consortium; CABG, coronary artery bypass graft; ECG, electrocardiogram; TIMI, thrombolysis in myocardial infarction; IABP, intra-aortic balloon pump; HF, heart failure; AUC, area under the curve; OMT, optimal medical therapy; GUSTO, Global Use of Strategies to Open Occluded Coronary Arteries; TLR, target-lesion revascularization.

Each definition of MI took into account different biomarker cut-offs based on the time of onset of symptoms from randomization, associated or not with pathological Q-waves or ECG changes. Troponin T or I may have been used for the diagnosis if CK-MB was not available. A definition for periprocedural MI is provided, discerning patients with already elevated enzymes from those without (*Table 1*).

#### Statistical analysis

For all main analyses, only variables with missingness <10% were considered. For categorical variables, an extra level to depict a missing value was created. Categorical variables were reported as counts and percentages and compared by either χ^2^ or Fisher's exact test. The distribution of continuous variables was evaluated through the Shapiro–Wilk test and reported as mean and standard deviation or median and interquartile range (IQR) for normal and non-normal distribution, respectively, and compared using either Student's *t*-test or Mann–Whitney *U*-test, as appropriate.

Time-to-first event was used to assess outcomes, and the analyses followed the intention-to-treat principle. Kaplan–Meier analysis was used to outline cumulative adverse events along with numbers at risk. To obtain the risk of each endpoint, one-stage, random-effects Cox proportional hazards regression models with shared frailty were conducted for comparisons, with treatment strategy assignment as the fixed component and the original RCT as the random component. Risk estimates were reported as hazard ratios (HR) and 95% confidence intervals (CI) with respective *P*-values and *P* for interaction between sex and treatment strategy. All tests were two-sided, with *P* < 0.05 considered significant. The analyses were performed in early and delayed cohorts with sex as predictor variable. As pre-specified, multivariable adjustment was based on the random effects Cox model taking into account age, diabetes mellitus, biomarkers status, and GRACE score. Further fixed-effect Cox proportional hazards models were computed as sensitivity analysis. As an exploratory analysis, the overall population was stratified by GRACE score. Low-risk and high-risk were defined by GRACE score ≤140 or >140, respectively. The analyses using random-effects Cox proportional hazards regression models were then performed within early and delayed cohorts, using sex as predictor variable.

The Cochrane Risk-of-Bias 2 (RoB 2) tool was used to evaluate potential sources of bias. Statistical between-trial heterogeneity was assessed through tau^2^ (*τ*^2^) and *I*^2^ tests. In case of high between-trial heterogeneity, additional analyses were performed to explore which of the included studies were sources of heterogeneity. Publication bias was assessed by Egger's regression test and visual assessment of funnel plots, using Trim-and-Fill method to correct the results for those endpoints suspected of publication bias. The analyses were conducted using R version 4.3.1 and R Studio version 1.4.1106 (R Foundation for Statistical Computing, Vienna, Austria).

## Results

Out of the 2232 results identified from literature source, 1292 titles and abstracts were screened. After exclusion of 1274 studies, 18 were evaluated for eligibility. Supplementary material online, *Table S2* provides a list of further 12 excluded studies. Six RCTs were included and provided IPD (see Supplementary material online, *Tables S3–S8*). The Leipzig Immediate vs. early and late PercutaneouS coronary Intervention triAl in NSTEMI (LIPSIA-NSTEMI) randomly assigned patients to three arms: immediate, early, and selectively invasive. Patients allocated to the latter were not included. A total of 6654 patients with complete follow-up data were analysed (see Supplementary material online, *Figure S1*). Three out of the six trials used a strategy of immediate invasive procedure for the early group (LIPSIA-NSTEMI, RIDDLE-NSTEMI, and ELISA-3), whereas for the other studies the median time to angiography in the early cohort consisted of a larger interval, up to 24 h after randomization. There was overlap between the IQR of the time to angiography in both the arms of LIPSIA-NSTEMI study and the early strategy of the other trials (see Supplementary material online, *Figure S2*). The median follow-up was 185 days (6 months).

### Heterogeneity test and publication bias

The between-trial heterogeneity was low for the primary endpoint (*τ*^2^ < 0.001, *I*^2^ 10%), death (*τ*^2^ 0.000, *I*^2^ 0%), stroke (*τ*^2^ < 0.000, *I*^2^ 0%), and major bleeding (*τ*^2^ < 0.004, *I*^2^ 0%). Conversely, MI and recurrent ischaemia were associated with high between-trial heterogeneity (MI: *τ*^2^ 0.356, *I*^2^ 62.9%; recurrent ischaemia: *τ*^2^ 0.139, *I*^2^ 56.8%). Therefore, for the endpoints MI and recurrent ischaemia, a two-stage meta-analysis was conducted, excluding the RCTs identified as the most influential and responsible for between-trial heterogeneity. The results indicated that the pooled effect remained non-significant, confirming consistency with the previous findings (see Supplementary material online, *Figures S5*–S10).

No significant sign of publication bias was detected for the primary endpoint (*P*-value = 0.726), death (*P*-value = 0.799), MI (*P*-value = 0.904), and recurrent ischaemia (*P*-value = 0.736). While Egger's test showed publication bias for stroke (*P*-value < 0.001) and major bleeding (*P*-value = 0.047), Trim-and-Fill correction did not identify any significant difference (stroke *P*-value = 0.549, major bleeding *P*-value = 0.379). Supplementary material online, *Figure S11* displays the funnel plots for visual estimation of the publication bias result reported for each endpoint.

#### Risk of bias

All trials had a low risk of bias. Considering the non-blinded allocation to different timing does not represent a high-risk feature, the authors judged the effect of assignment to intervention section to be low risk. However, it should be taken into account that blinding the treatment strategy was not possible since patients and medical staff were aware of the timing (see Supplementary material online, *Figure S12*).

#### Baseline characteristics

Out of the total 6654 patients, 2257 (33.9%) were female with a median age of 69 years (IQR 60–76), significantly higher than male patients (64.5 years, IQR 55–72, *P* < 0.001). There were significant differences in baseline characteristics between females and males as shown in *Table 2*. Fewer females were former or current smokers (21.7 vs. 34.2%, *P* < 0.001) and had a past medical history positive for previous MI (14.5 vs. 20.5%, *P* < 0.001), coronary artery bypass grafting (CABG) (4.4. vs. 8.4%, *P* < 0.001), and percutaneous coronary intervention (PCI) (11.3 vs. 16.9%, *P* < 0.001) compared with males. In contrast, more females had hypertension (68 vs. 59.3%, *P* < 0.001) and diabetes mellitus (27.2 vs. 21.2%, *P* < 0.001) than males. Higher GRACE scores were identified in females compared with males [136 (IQR 117–157) vs. 131 (IQR 113–151), *P* < 0.001]. Non-obstructive coronaries were reported more frequently in females (29.3 vs. 12.8%, *P* < 0.001). Compared with females, males had a higher incidence of three-vessel disease (19.6 vs. 14.4%, *P* < 0.001) and left main involvement (8.6 vs. 5.4%, *P* < 0.001). Significant differences in secondary prevention medications at discharge were found, with females more frequently receiving calcium channel blockers (19.4 vs. 16.1%, *P* = 0.003) and angiotensin-receptor blockers (11.3 vs. 9.4%, *P* = 0.049), whereas males were more often prescribed beta-blockers (70.5 vs. 66%, *P* < 0.001), angiotensin-converting enzyme inhibitors (48.8 vs. 45.4%, *P* = 0.006), and statins (75.5 vs. 72.3%, *P* < 0.001) (*Table 2*).

**Table 2:** Baseline characteristics in female and male patients

Variables	Female patients	Male patients	*P*-value
	*n* = 2257	*n* = 4397	
Demographic data and clinical measures			
Age, years [IQR]	69 [60, 76]	64.5 [55, 72.1]	<0.001
BMI, kg m^−2^[IQR]	26.1 [23.4, 29.4]	26.9 [24.6, 29.8]	<0.001
GRACE score [IQR]	136 [117, 157]	131 [113, 151]	<0.001
HR [IQR]	78 [68, 90]	75 [65, 87]	<0.001
Systolic BP, mmHg [IQR]	141 [127, 160]	140 [125, 157]	0.009
Creatinine, µmol/L [IQR]	67 [58, 80]	83 [72, 96]	<0.001
Past medical history			
Former or current smoking, (%)	489 (21.7)	1504 (34.2)	<0.001
Missing data, (%)	0 (0.0)	3 (0.1)	
Previous MI, (%)	328 (14.5)	903 (20.5)	<0.001
Previous CABG, (%)	100 (4.4)	371 (8.4)	<0.001
Previous PCI, (%)	255 (11.3)	743 (16.9)	<0.001
Missing data, (%)	1 (0.0)	3 (0.1)	
Previous stroke, *n* (%)	144 (6.4)	307 (7.0)	0.448
Missing data, (%)	128 (5.7)	271 (6.2)	
Hypertension, *n* (%)	1535 (68.0)	2607 (59.3)	<0.001
Missing data, (%)	1 (0.0)	3 (0.1)	
Diabetes mellitus, *n* (%)	615 (27.2)	931 (21.2)	<0.001
Medical therapy at discharge			
Aspirin, *n* (%)	1860 (82.4)	3870 (88.0)	<0.001
Missing data, (%)	100 (4.4)	189 (4.3)	
Clopidogrel, *n* (%)	1087 (48.2)	2416 (54.9)	<0.001
Missing data, (%)	99 (4.4)	192 (4.4)	
Ticagrelor, *n* (%)	350 (15.5)	807 (18.4)	0.008
Missing data, (%)	226 (10.0)	461 (10.5)	
Prasugrel, *n* (%)	7 (0.3)	34 (0.8)	0.053
Missing data, *n* (%)	1326 (58.8)	2526 (57.4)	
BBs, *n* (%)	1489 (66.0)	3098 (70.5)	<0.001
Missing data, *n* (%)	276 (12.2)	562 (12.8)	
ACEis, *n* (%)	1025 (45.4)	2147 (48.8)	0.006
Missing data, *n* (%)	277 (12.3)	567 (12.9)	
ARBs, *n* (%)	254 (11.3)	415 (9.4)	0.049
Missing data, *n* (%)	382 (16.9)	793 (18.0)	
CCBs, *n* (%)	438 (19.4)	706 (16.1)	0.003
Missing data, *n* (%)	379 (16.8)	782 (17.8)	
Statin, *n* (%)	1632 (72.3)	3321 (75.5)	<0.001
Missing data, *n* (%)	275 (12.2)	562 (12.8)	
Procedural characteristics			
Involved vessels, *n* (%)			<0.001
0	662 (29.3)	565 (12.8)	
1	636 (28.2)	1418 (32.2)	
2	422 (18.7)	1044 (23.7)	
3	326 (14.4)	863 (19.6)	
LM	123 (5.4)	377 (8.6)	
Missing data, *n* (%)	88 (3.9)	130 (3.0)	
Time to angiography, hours [IQR]	20 [3.9, 50.7]	20 [3.7, 51]	0.732
Time to PCI, hours [IQR]	12.4 [2.4, 48.1]	14.4 [3, 49.7]	0.137
Time to CABG, days [IQR]	10.3 [6, 15]	8.5 [1.2, 13.1]	0.002
Treatment strategy, *n* (%)			<0.001
Medical	927 (41.1)	973 (22.1)	
PCI	1024 (45.4)	2591 (58.9)	
CABG	224 (9.9)	703 (16.0)	
No angio	82 (3.6)	129 (2.9)	
Missing data	0 (0.0)	1(0.0)	
Hospital stay, days [IQR]	4 [3, 8]	4 [3, 9]	0.545

Abbreviations. BMI, body mass index; GRACE, Global Registry of Acute Coronary Events; HR, heart rate; BP, blood pressure; MI, myocardial infarction; BBs, beta-blockers; ARBs, angiotensin-receptor inhibitors; CCBs, calcium channel blockers; ACEi, angiotensin-converting enzyme inhibitors; CABG, coronary artery bypass graft; IQR, interquartile range; *n*, number; PCI, percutaneous coronary intervention.

#### Sex differences in outcomes in the early strategy

**Figure 1:**
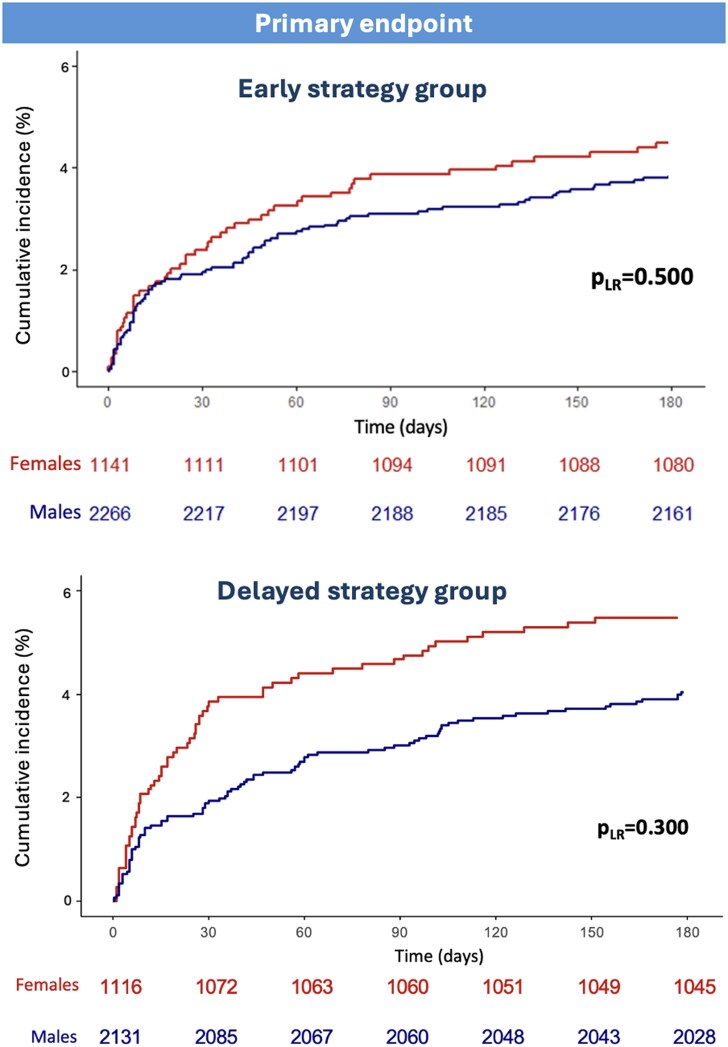
Primary endpoint events in patients undergoing early and delayed invasive strategy stratified by sex. Abbreviations: *p*_LR_ = *P*-log rank.

**Figure 2:**
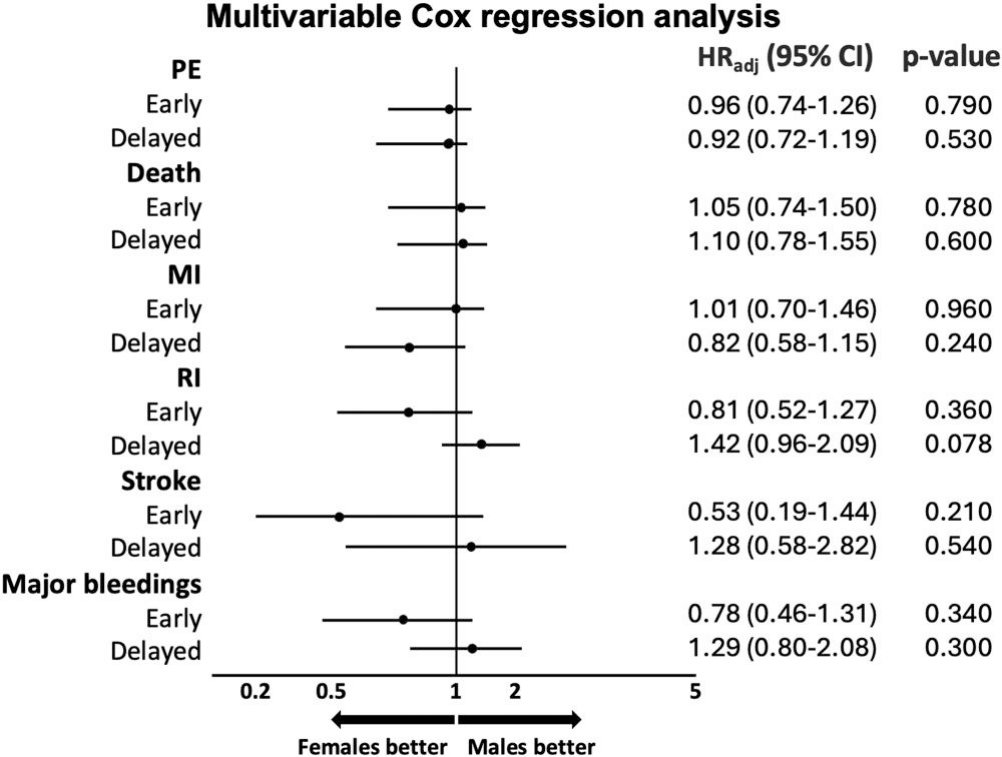
Multivariable Cox regression analysis using random effect in the early and delayed invasive strategy groups stratified by sex. Forest plots show multivariable Cox regression analysis using random effect, adjusted for age, diabetes mellitus, biomarkers status, and Global Registry of Acute Coronary Events score. Abbreviations: PE, primary endpoint; MI, myocardial infarction; RI, recurrent ischaemia; HRadj, hazard ratio adjusted; CI, confidence interval; GRACE, Global Registry of Acute Coronary Events.

Among patients allocated to the early invasive strategy, no difference in the risk and incidence of the primary endpoint (females: 7.5% vs. males: 6.9%, HR 1.08, 95% CI 0.83–1.41, *P* = 0.560) was found using random-effects Cox regression models at both univariable and multivariable analysis (*Figure 1* and *Table 3*). There was also no difference in the individual risk of all-cause death, MI, recurrent ischaemia, stroke, and major bleeding (*Table 3*, Supplementary material online, *Figure S3*, *Figure 2*). Similar findings were obtained using fixed-effect model (see Supplementary material online, *Table S9*).

**Table 3:** Comparison between females and males in the early and delayed invasive strategy groups

	Early strategy (*n* = 3407)						Delayed strategy (*n* = 3247)						
Endpoints	Females (*n* = 1141)	Males (*n* = 2266)	HR^a^ (95% CI)	*P*-value	HR adjusted^b^	*P*-value	Females (*n* = 1116)	Males (*n* = 2131)	HR^a^ (95% CI)	*P*-value	HR adjusted^b^	*P*-value	*P*-interaction
Primary endpoint	86 (7.5)	157 (6.9)	1.08 (0.83–1.41)	0.560	0.96 (0.74–1.26)	0.790	103 (9.2)	178 (8.4)	1.12 (0.88–1.43)	0.350	0.92 (0.72–1.19)	0.530	0.850
All-cause death	51 (4.5)	86 (3.8)	1.18 (0.83–1.66)	0.360	1.05 (0.74–1.50)	0.780	61 (5.5)	86 (4.0)	1.38 (0.99–1.91)	0.056	1.10 (0.78–1.55)	0.600	0.510
Myocardial infarction	46 (4)	79 (3.5)	1.15 (0.80–1.66)	0.440	1.01 (0.70–1.46)	0.960	53 (4.7)	105 (4.9)	0.97 (0.70–1.35)	0.860	0.82 (0.58–1.15)	0.240	0.490
Recurrent ischaemia	32 (2.8)	80 (3.5)	0.84 (0.56–1.27)	0.420	0.81 (0.52–1.27)	0.360	51 (4.6)	69 (3.2)	1.52 (1.06–2.19)	0.023	1.42 (0.96–2.09)	0.078	0.011
Stroke	5 (0.4)	17 (0.8)	0.56 (0.21–1.52)	0.260	0.53 (0.19–1.44)	0.210	12 (1.1)	16 (0.8)	1.46 (0.69–3.08)	0.320	1.28 (0.58–2.82)	0.540	0.150
Major bleeding	22 (1.9)	51 (2.3)	0.84 (0.51–1.38)	0.480	0.78 (0.46–1.31)	0.340	41 (3.7)	44 (2.1)	1.88 (1.22–2.87)	0.004	1.29 (0.80–2.08)	0.300	0.019

CI, confidence interval; HR, hazard ratio; *n*, number. GRACE, Global Registry of Acute Coronary Events.

^a^HR and 95% CI for the comparison between early and delayed strategy using random effect Cox model to adjust for within-study clustering. Univariable unadjusted model for treatment strategy.

^b^HR adjusted for age, diabetes mellitus, biomarkers status, and GRACE score.

#### Sex differences in outcomes in the delayed strategy

Among patients allocated to the delayed invasive strategy, no difference in the risk and incidence of the primary endpoint (females: 9.2% vs. males: 8.4%, HR 1.12, 95% CI 0.88–1.43, *P* = 0.350) was found (*Table 3* and *Figure 1*). There was also no difference in the risk of all-cause death, MI and stroke (see Supplementary material online, *Figure S4*).

**Figure 3:**
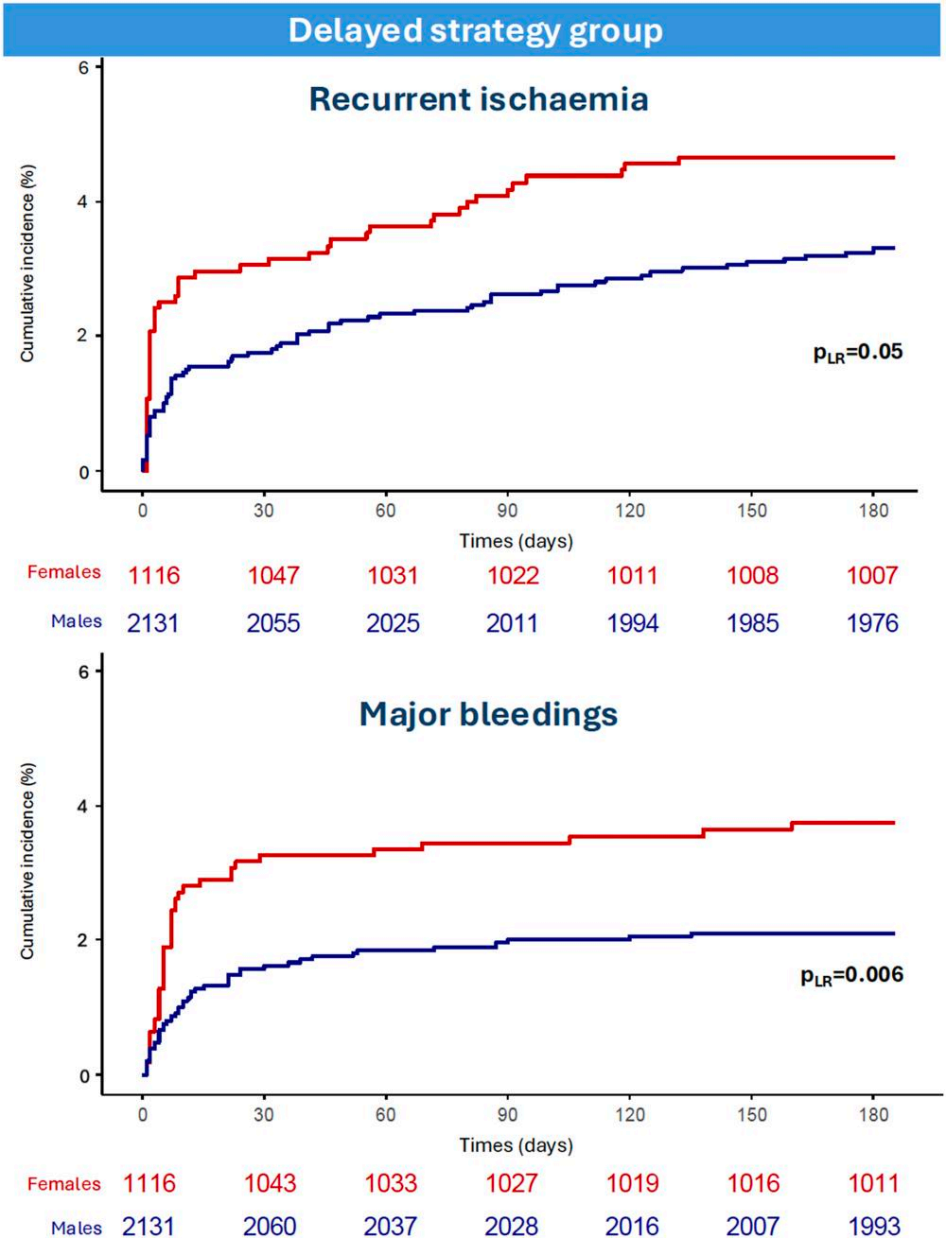
Recurrent ischaemia and major bleeding events in patients undergoing delayed invasive strategy stratified by sex. Abbreviations: *p*_LR_ = *p*-log rank.

Female sex vs. male sex was associated with increased risk and incidence of recurrent ischaemia (4.6 vs. 3.2%, HR 1.52, 95% CI 1.06–2.19, *P* = 0.023) and major bleeding (3.7 vs. 2.1%, HR 1.88, 95% CI 1.22–2.87, *P* = 0.004) using univariable analysis (*Table 3* and *Figure 3*). Multivariable analysis did not confirm these differences as statistically significant (*Table 3* and *Figure 2*). Tests for interaction between sex and treatment strategy were statistically significant for recurrent ischaemia and major bleeding (*P*-value for interaction 0.011 and 0.019, respectively) (*Table 3*). Similar findings were obtained using fixed-effect model (see Supplementary material online, *Table S9*).

#### Sex differences within early and delayed strategy groups stratified by Global Registry of Acute Coronary Events score

Risk stratification using GRACE score was available in 95.8% (*n* = 6372) of patients. No significant difference in the primary and secondary endpoints between females and males was reported in patients undergoing early invasive strategy, regardless of the level of risk.

Among low-risk patients allocated to delayed invasive strategy, female sex vs. male sex was associated with increased incidence and the risk of major bleeding (3.2 vs. 1.4%, HR 2.34, 95% CI 1.23–4.46, *P* = 0.010) (see Supplementary material online, *Table S10*). Among high-risk patients allocated to delayed strategy, female sex vs. male sex was associated with an increased incidence and risk of recurrent ischaemia (5.8 vs. 3.9%, HR 1.69, 95% CI 1.01–2.83, *P* = 0.048) (see Supplementary material online, *Table S11*). These findings were confirmed by the analyses performed using fixed-effect model (see Supplementary material online, *Tables S12* and *S13*).

## Discussion

Among patients undergoing early or delayed invasive strategy for the management of NSTE-ACS, there were no differences in the risk of primary endpoint between females and males. Among patients undergoing delayed invasive strategy, female sex was associated with increased risk of recurrent ischaemia and major bleeding using univariable analysis but not using multivariable analysis.

This IPD meta-analysis confirms that female NSTE-ACS patients present with different cardiovascular risk factors, past medical history, and are treated with different secondary prevention medications at discharge compared with male patients as well as a different revascularization strategy, a finding consistent with prior studies.^24–28^ In both early and delayed groups, females had a slightly higher composite risk of all-cause mortality and MI compared with males, although this difference did not reach statistical significance.

The variation in the timing of early intervention across the included RCTs obscures the true impact of treatment on new MI occurrence. Furthermore, the significant difference in the rate of revascularization (PCI or CABG) females and males underwent (55.3 vs. 74.9%, respectively), as well as the differences in multivessel disease, may have impacted on this result. A meta-regression revealed that a wider time interval between early and delayed treatment is associated with lower risk of new MI in patients allocated to early invasive strategy. This association was shown to be stronger in studies with higher PCI rates, indicating a positive effect of early treatment in a population suffering from a more severe coronary artery disease (CAD).^29^

In the current analysis, the difference in the median time to PCI between early and delayed groups in both sexes corresponded to 45.4 h, equivalent to approximately half of the ISAR-COOL trial, excluded from the present meta-analysis due to the long time period in the delayed group.^30^ We reported three-vessel disease and left main involvement more frequently in men compared with women and, as a consequence, a significantly higher percentage of men underwent revascularization compared with women, resulting in stabilization of such a great number of severe CAD including unstable/significant plaques in male patients.

The univariable Cox regression analysis confirmed the association between female sex and the risk of recurrent ischaemia and major bleeding among patients receiving delayed strategy. In NSTE-ACS, ruling out epicardial CAD is the first step towards establishing a diagnosis of myocardial infarction with non-obstructive coronary arteries (MINOCA) occurring more frequently in females.^31^ In our study, females had more non-obstructive CAD (29.3 vs. 12.8%, *P* < 0.001) than males. This spectrum encompasses a heterogeneous group of underlying causes, including both extracardiac and cardiac origins, with the latter arising from coronary or non-coronary mechanisms. Therefore, a prompt pathophysiological characterization in the female population with an early invasive strategy, aims to control symptoms and prevent recurrent ischaemia, but also to tailor pharmacological therapy to address specific pathophysiological mechanisms (such as vasospastic angina and coronary microvascular dysfunction) and decrease bleeding risk.

Female sex is often associated with criteria defining high bleeding risk, such as older age, kidney disease, and anaemia.^32,33^ In our study, among patients undergoing delayed strategy females experience bleeding complications more frequently than males. The multivariable analysis did not confirm the association between female sex and the risk of recurrent ischaemia and major bleedings in patients receiving delayed invasive strategy. This finding suggests the potential influence of confounding variables not fully accounted for in the present analysis. The time interval between the onset of symptoms and presentation in hospital has been demonstrated to be longer in females compared with males.^34^ Females experience delays secondary to lack of awareness, socio-economic barriers, and underestimated risk. These factors, in addition to the older age and higher GRACE score, should be taken into account and added to the prolonged time in the delayed invasive strategy arm, leading to recurrent symptoms during the follow-up. Moreover, it is also important to take into consideration the role of concomitant conditions rather than an independent biological association between female sex and haemorrhagic risk.^35^

We showed that in patients with a higher baseline ischaemic risk profile (defined by GRACE score >140) in whom invasive management was delayed, female sex was associated with higher risk of recurrent ischaemia (see Supplementary material online, *Tables S10* and *S11*). On the other hand, in low-risk patients (GRACE score ≤140) and a delayed procedure, females were more prone to develop major bleeding. These findings underscore a potentially differential role of baseline risk stratification for male and female NSTE-ACS patients, with a need to define coronary anatomy early in females to tailor treatment and initiate further diagnostic tests if needed, particularly given the fact that nearly one-third of females have non-obstructive CAD.

Regardless of risk stratification, the increased risk of recurrent ischaemia in females compared with males undergoing delayed strategy does not correlate with subsequent higher rate of MI events. This is supported by a large meta-analysis which reported a significant reduction in the risk of recurrent ischaemia among NSTE-ACS patients using an early strategy compared with a delayed strategy.^36^ Taken altogether, it seems fair to speculate that the higher proportion of females with non-obstructive disease may reflect that recurrent ischaemia could be caused by other pathophysiological entities (such as coronary microvascular dysfunction).

### Strengths and limitations

This *post-hoc* analysis provides important findings given the availability of patient-level data, which allowed for more complex statistical approaches and greater statistical power to investigate interactions between treatment strategies and covariates. However, this study has several limitations. Heterogeneity across the studies with respect to inclusion criteria, timing of invasive strategy, endpoint definitions, and follow-up might have had an impact on the results. The definitions of recurrent ischaemia differed between trials, and data on major bleeding were available in only four studies. There was lack of universal definition of non-fatal MI across the trials. Risk stratification was performed using GRACE score, since data on high-sensitivity c Troponin and ST-elevation were missing in five studies. Previous studies have demonstrated sex-specific limitations of the GRACE 2.0 score, exemplified by a systematic underestimation of in-hospital mortality risk in females.^5^ Data on dynamic ST-segment/T waves changes was not available. Variables regarding procedural characteristics (access site, anticoagulation dose, and procedural time) and comorbidities to better investigate sex-based differences are lacking, as are data on time interval between symptom onset and presentation. Furthermore, relevant clinical covariates, such as kidney failure and antithrombotic therapy, were not considered in the multivariable analysis, which was limited to variables pre-specified in PROSPERO. The findings should be interpreted with caution due to the limitations of *post-hoc* analyses, which may not fully exclude bias or confounding factors related to sex-based comparisons. Two out of the six endpoints, MI and recurrent ischaemia, were associated with high between-trial heterogeneity, while stroke and major bleeding may have been influenced by publication bias. However, Trim-and-Fill correction provided consistent outcomes. Lastly, the median follow-up was 185 days, and further long-term evidence is needed.

## Conclusion

In this IPD meta-analysis, no sex-related differences in the composite of all-cause death or MI were identified among NSTE-ACS patients undergoing early and delayed invasive management. Among patients treated with a delayed invasive strategy, female sex was associated with increased risk of recurrent ischaemia and major bleeding using univariable analysis but not using multivariable analysis (*Graphical abstract*). The present *post-hoc* analysis should encourage physicians to take into account and expect different adverse clinical outcomes between sexes in cases where a delayed invasive strategy is adopted. There is a need to focus on subsets of patients who are typically under-represented and under-investigated in most RCTs, such as women with cardiovascular disease, highlighting the impact of female biology on specific treatments to overcome delays in management. Further large-scale research using contemporary treatment strategies is needed to investigate sex-related differences in long-term clinical outcomes and to inform optimal care among females.

## Supplementary Material

oeaf059_Supplementary_Data

## Data Availability

Individual participant-level data used for this study are not publicly available, because they contain protected patient health information. Requests for data access should be directed to the corresponding author via email.
