# Supplementary material for: Sex differences based on the timing of invasive management among patients with non-ST-elevation acute coronary syndrome: an individual patient data meta-analysis
Source: Eur Heart J Open. 2025 May 17;5(3):oeaf059. doi: 10.1093/ehjopen/oeaf059 (PMC12202155; doi:10.1093/ehjopen/oeaf059)
Supplement: oeaf059_Supplementary_Data [file oeaf059_supplementary_data.docx]

**Supplementary materials**

**Supplementary methods**

**Search Algorithm**

The following terms were used without language and date restriction (up to November 2023): (ACS or NSTEMI or NSTEACS or ‘acute coronary syndrome’ or ‘non-ST elevation myocardial infarction’ or ‘non-ST elevation acute coronary syndrome’) AND (‘invasive’ or ‘angiography’ or ‘strategy’ or ‘intervention’ or ‘angioplasty’ or PCI or ‘percutaneous coronary intervention’ or ‘revascularisation’) AND (‘timing’ or ‘early’ or ‘immediate’ or ‘standard’ or ‘delayed’ or ‘late’).

**Supplementary table 1. PRISMA-IPD checklist items to include in a systematic review or meta-analysis of individual participant data (IPD)**

| **PRISMA-IPD**  **Section/topic** | **Item**  **No** | **Checklist item** | **Reported on page** |
| --- | --- | --- | --- |
| **Title** | | | |
| Title | 1 | Identify the report as a systematic review and meta-analysis of individual participant data. | Page 1 |
| **Abstract** | | | |
| Structured summary | 2 | Provide a structured summary including as applicable: | Page 3 |
|  |  | **Background**: state research question and main objectives, with information on participants, interventions, comparators and outcomes. |  |
|  |  | **Methods**: report eligibility criteria; data sources including dates of last bibliographic search or elicitation, noting that IPD were sought; methods of assessing risk of bias. |  |
|  |  | **Results**: provide number and type of studies and participants identified and number (%) obtained; summary effect estimates for main outcomes (benefits and harms) with confidence intervals and measures of statistical heterogeneity. Describe the direction and size of summary effects in terms meaningful to those who would put findings into practice. |  |
|  |  | **Discussion:** state main strengths and limitations of the evidence, general interpretation of the results and any important implications. |  |
|  |  | **Other:** report primary funding source, registration number and registry name for the systematic review and IPD meta-analysis. |  |
| **Introduction** | | | |
| Rationale | 3 | Describe the rationale for the review in the context of what is already known. | Pages 5-6 |
| Objectives | 4 | Provide an explicit statement of the questions being addressed with reference, as applicable, to participants, interventions, comparisons, outcomes and study design (PICOS). Include any hypotheses that relate to particular types of participant-level subgroups. | Pages 5-6 |
| **Methods** | | | |
| Protocol and registration | 5 | Indicate if a protocol exists and where it can be accessed. If available, provide registration information including registration number and registry name. Provide publication details, if applicable. | Page 6 |
| Eligibility criteria | 6 | Specify inclusion and exclusion criteria including those relating to participants, interventions, comparisons, outcomes, study design and characteristics (e.g. years when conducted, required minimum follow-up). Note whether these were applied at the study or individual level i.e. whether eligible participants were included (and ineligible participants excluded) from a study that included a wider population than specified by the review inclusion criteria. The rationale for criteria should be stated. | Page 6 |
| Identifying studies - information sources | 7 | Describe all methods of identifying published and unpublished studies including, as applicable: which bibliographic databases were searched with dates of coverage; details of any hand searching including of conference proceedings; use of study registers and agency or company databases; contact with the original research team and experts in the field; open adverts and surveys. Give the date of last search or elicitation. | Page 6 |
| Identifying studies - search | 8 | Present the full electronic search strategy for at least one database, including any limits used, such that it could be repeated. | Supplementary figure 1  Page 6 |
| Study selection processes | 9 | State the process for determining which studies were eligible for inclusion. | Page 6 |
| Data collection processes | 10 | Describe how IPD were requested, collected and managed, including any processes for querying and confirming data with investigators. If IPD were not sought from any eligible study, the reason for this should be stated (for each such study). | Pages 6-7  Supplementary figure 1 |
|  |  | If applicable, describe how any studies for which IPD were not available were dealt with. This should include whether, how and what aggregate data were sought or extracted from study reports and publications (such as extracting data independently in duplicate) and any processes for obtaining and confirming these data with investigators. |  |
| Data items | 11 | Describe how the information and variables to be collected were chosen. List and define all study level and participant level data that were sought, including baseline and follow-up information. If applicable, describe methods of standardising or translating variables within the IPD datasets to ensure common scales or measurements across studies. | Pages 6-7 |
| IPD integrity | A1 | Describe what aspects of IPD were subject to data checking (such as sequence generation, data consistency and completeness, baseline imbalance) and how this was done. | Pages 6-7 |
| Risk of bias assessment in individual studies. | 12 | Describe methods used to assess risk of bias in the individual studies and whether this was applied separately for each outcome. If applicable, describe how findings of IPD checking were used to inform the assessment. Report if and how risk of bias assessment was used in any data synthesis. | Page 9  Supplementary figure 6 |
| Specification of outcomes and effect measures | 13 | State all treatment comparisons of interests. State all outcomes addressed and define them in detail. State whether they were pre-specified for the review and, if applicable, whether they were primary/main or secondary/additional outcomes. Give the principal measures of effect (such as risk ratio, hazard ratio, difference in means) used for each outcome. | Pages 7-8  Table 1 |
| Synthesis methods | 14 | Describe the meta-analysis methods used to synthesise IPD. Specify any statistical methods and models used. Issues should include (but are not restricted to):   - Use of a one-stage or two-stage approach. - How effect estimates were generated separately within each study and combined across studies (where applicable). - Specification of one-stage models (where applicable) including how clustering of patients within studies was accounted for. - Use of fixed or random effects models and any other model assumptions, such as proportional hazards. - How (summary) survival curves were generated (where applicable). - Methods for quantifying statistical heterogeneity (such as I^2^ and τ^2^). - How studies providing IPD and not providing IPD were analysed together (where applicable). - How missing data within the IPD were dealt with (where applicable). | Pages 7-8 |
| Exploration of variation in effects | A2 | If applicable, describe any methods used to explore variation in effects by study or participant level characteristics (such as estimation of interactions between effect and covariates). State all participant-level characteristics that were analysed as potential effect modifiers, and whether these were pre-specified. | Pages 7-8 |
| Risk of bias across studies | 15 | Specify any assessment of risk of bias relating to the accumulated body of evidence, including any pertaining to not obtaining IPD for particular studies, outcomes or other variables. | Page 10 |
| Additional analyses | 16 | Describe methods of any additional analyses, including sensitivity analyses. State which of these were pre-specified. | Pages 8-9 |
| **Results** | | | |
| Study selection and IPD obtained | 17 | Give numbers of studies screened, assessed for eligibility, and included in the systematic review with reasons for exclusions at each stage. Indicate the number of studies and participants for which IPD were sought and for which IPD were obtained. For those studies where IPD were not available, give the numbers of studies and participants for which aggregate data were available. Report reasons for non-availability of IPD. Include a flow diagram. | Page 9 |
| Study characteristics | 18 | For each study, present information on key study and participant characteristics (such as description of interventions, numbers of participants, demographic data, unavailability of outcomes, funding source, and if applicable duration of follow-up). Provide (main) citations for each study. Where applicable, also report similar study characteristics for any studies not providing IPD. | Supplementary tables 3-8 |
| IPD integrity | A3 | Report any important issues identified in checking IPD or state that there were none. | Pages 9-10 |
| Risk of bias within studies | 19 | Present data on risk of bias assessments. If applicable, describe whether data checking led to the up-weighting or down-weighting of these assessments. Consider how any potential bias impacts on the robustness of meta-analysis conclusions. | Non applicable |
| Results of individual studies | 20 | For each comparison and for each main outcome (benefit or harm), for each individual study report the number of eligible participants for which data were obtained and show simple summary data for each intervention group (including, where applicable, the number of events), effect estimates and confidence intervals. These may be tabulated or included on a forest plot. | Non applicable |
| Results of syntheses | 21 | Present summary effects for each meta-analysis undertaken, including confidence intervals and measures of statistical heterogeneity. State whether the analysis was pre-specified, and report the numbers of studies and participants and, where applicable, the number of events on which it is based. | Non applicable |
|  |  | When exploring variation in effects due to patient or study characteristics, present summary interaction estimates for each characteristic examined, including confidence intervals and measures of statistical heterogeneity. State whether the analysis was pre-specified. State whether any interaction is consistent across trials. |  |
|  |  | Provide a description of the direction and size of effect in terms meaningful to those who would put findings into practice. |  |
| Risk of bias across studies | 22 | Present results of any assessment of risk of bias relating to the accumulated body of evidence, including any pertaining to the availability and representativeness of available studies, outcomes or other variables. | Page 9 |
| Additional analyses | 23 | Give results of any additional analyses (e.g. sensitivity analyses). If applicable, this should also include any analyses that incorporate aggregate data for studies that do not have IPD. If applicable, summarise the main meta-analysis results following the inclusion or exclusion of studies for which IPD were not available. | Supplementary tables 10-14 |
| **Discussion** | | | |
| Summary of evidence | 24 | Summarise the main findings, including the strength of evidence for each main outcome. | Page 13 |
| Strengths and limitations | 25 | Discuss any important strengths and limitations of the evidence including the benefits of access to IPD and any limitations arising from IPD that were not available. | Pages 16-17 |
| Conclusions | 26 | Provide a general interpretation of the findings in the context of other evidence. | Page 17 |
| Implications | A4 | Consider relevance to key groups (such as policy makers, service providers and service users). Consider implications for future research. | Page 17 |
| **Funding** | | | |
| Funding | 27 | Describe sources of funding and other support (such as supply of IPD), and the role in the systematic review of those providing such support. | Page 18 |

**Supplementary table 2. Excluded studies**

| **Title** | **First author** | **Journal and Year** | **PMID** | **Exclusion reason** |
| --- | --- | --- | --- | --- |
| Optimal timing of coronary stenting in unstable angina patients | Shen W | Chin Med J (Engl)  2001 | 11779437 | Unstable angina only |
| Evaluation of prolonged antithrombotic pretreatment ("cooling-off" strategy) before intervention in patients with unstable coronary syndromes: a randomized controlled trial | Neumann FJ | JAMA  2003 | 14506118 | No response |
| Immediate versus deferred coronary angioplasty in non-ST-segment elevation acute coronary syndromes | Riezebos RK | Heart  2009 | 19098058 | Randomised after angiography |
| Immediate vs delayed intervention for acute coronary syndromes: a randomized clinical trial | Montalescot G | JAMA  2009 | 19724041 | No response |
| Comparison of immediate vs early invasive strategy in patients with first acute non-ST-elevation myocardial infarction | Sciahbasi A | Clin Cardiol  2010 | 20960541 | Small trial with few female participants |
| Outcome of patients with non-ST segment elevation acute coronary syndrome undergoing early or delayed intervention | Zhang J | Chinese  2010 | 21176626 | Unable to contact author |
| Influence of the Timing of Percutaneous Coronary Intervention on Clinical Outcomes in Non-ST-Elevation Myocardial Infarction | Tekin K | Korean Circ J  2013 | 24363747 | Small trial with few female participants |
| Evaluation of early interventional treatment opportunity of the elderly & high-risk patients with non-ST segment elevation acute myocardial infarction | Liu Z | Pak J Med Sci  2015 | 26648985 | Small trial with few female participants |
| Early invasive strategy in high-risk acute coronary syndrome without ST-segment elevation. The Sisca randomized trial | Reuter PG | Int J Cardiol.  2015 | 25596468 | Comparison to selective invasive treatment |
| Comparison of Acute Versus Subacute Coronary Angiography in Patients with NON-ST-Elevation Myocardial Infarction (from the NONSTEMI Trial) | Rasmussen MB | Am J Cardiol  2019 | 31324357 | Pre-hospital randomisation |
| Optimal Timing of Intervention in NSTE-ACS Without Pre-Treatment: The EARLY Randomized Trial | Lemesle G | JACC Cardiovasc Interv  2020 | 32327087 | No response |
| An immediate or early invasive strategy in non-ST-elevation acute coronary syndrome: The OPTIMA-2 randomized controlled trial | Fagel ND | Am Heart J  2021 | 33422517 | No response |

**Supplementary table 3. Key information trial**

**van’t Hof et al., 2003 (ELISA)**

| **Principal Investigator** | A W J van 't Hof |
| --- | --- |
| **Country** | The Netherlands |
| **Study Period** | April 2000 - December 2001 |
| **Study Design** | Randomised controlled study.  ***Early group:*** angiography within 12 h without tirofiban pre-treatment.  ***Late group:*** angiography after 12 h with tirofiban pre-treatment. Maintenance of tirofiban infusion was continued beyond angiography for at least 12 h in case of PCI. |
| **Inclusion Criteria** | Presence of at least two of the following criteria: unstable angina pectoris, not induced by a non-cardiac condition, with the last episode of pain within the preceding 24 h, ST-segment depression >1 mm or a positive Tn T (>0.05 ng/ml) on admission or 3 h later. |
| **Exclusion Criteria** | Patients with negative T waves only, over 80 years of age, who had a PCI within the preceding 6 months and with renal failure; contra-indication to treatment with a GPIIb/IIIa inhibitor; patients in Killip class 3 or 4. |
| **Follow up visits** | 30 days post-randomisation |
| **Outcomes** | ***Primary*:** Enzymatic infarct size.  ***Secondary*:** Total death and recurrent MI at 30-day follow-up; major bleedings. |
| **Ethical Review** | The study was approved by the ethics committees of the participating hospitals. |
| **Overall bias assessment** | Low risk |
| PCI: percutaneous coronary intervention; Tn: troponin; h: hours; MI: myocardial infarction. | |

**Supplementary table 4. Key information trial**

**Badings et al., 2013 (ELISA-3)**

| **Principal Investigator** | A W J van 't Hof |
| --- | --- |
| **Country** | The Netherlands |
| **Study Period** | July 2007 - June 2012 |
| **Study Design** | Randomised, open, multicentre study.  ***Early:*** angiography performed as soon as possible but within 12 h of randomisation.  ***Delayed:*** angiography performed no sooner than 48 h after randomisation unless clinical instability or recurrent ischaemia despite OMT warranted emergency angiography. |
| **Inclusion Criteria** | Patients hospitalised with ischaemic chest pain or dyspnoea at rest, with the last episode occurring 24 h or less before randomisation, and at least two out of three of the following high-risk characteristics: (1) extensive myocardial ischaemia on ECG (new cumulative ST depression >5 mm or temporary ST-segment elevation in two contiguous leads <30 min), (2) elevated biomarkers (Tn T >0.10 µg/l or myoglobin >150 µg/l) or elevated CKMB fraction (>6% of total CK), (3) age > 65 years. |
| **Exclusion Criteria** | Persistent ST-segment elevation, symptoms of ongoing myocardial ischaemia despite OMT, contraindication for diagnostic angiography, active bleeding, cardiogenic shock, acute posterior infarction, and life expectancy less than one year. |
| **Follow up visits** | 30 days post-randomisation |
| **Outcomes** | ***Primary*:** Combined incidence of all cause death, reinfarction and/or recurrent ischaemia at 30-day follow-up.  ***Secondaries*:** Enzymatic infarct size as assessed by a single cardiac Tn T (72-96 h after admission or at discharge); percentage of patients without a rise in CKMB during admission; bleeding complications. |
| **Ethical Review** | The study was approved by the ethics committees of the participating hospitals. |
| **Overall bias assessment** | Low risk |
| Tn: troponin; h: hours; ECG: electrocardiogram; CKMB: creatin kinase myocardial band. | |

**Supplementary table 5. Key information trial**

**Mehta et al., 2009 (TIMACS)**

| **Principal Investigator** | S Mehta |
| --- | --- |
| **Country** | Canada |
| **Study Period** | April 2003 - June 2008 |
| **Study Design** | Randomized, parallel-group, multicentre trial.  ***Early:*** ICA performed as rapidly as possible and within 24 h after randomization.  ***Delayed:*** ICA performed after a minimum delay of 36 h after randomization. |
| **Inclusion Criteria** | Patients presented with UA or NSTEMI within 24 h after the symptoms onset and with two of the following three criteria of increased risk: an age of 60 years or older, cardiac biomarkers above the ULN range, or ECG results compatible with ischaemia (i.e., ST-segment depression of ≥1 mm or transient ST-segment elevation or T-wave inversion of >3 mm). |
| **Exclusion Criteria** | Patients not suitable for coronary revascularization, comorbidity with life expectancy less than 6 months or <21 years of age. The initial 1,633 patients randomized were subjected to additional exclusion criteria associated with the OASIS 5 trial including: a contraindication to LMWH, severe renal insufficiency (creatinine >3 g/dL), haemorrhagic stroke within the past 12 months or indication for anticoagulation other than ACS. |
| **Follow up visits** | 6 months post-randomisation |
| **Outcomes** | ***Primary*:** First occurrence of the composite of death, new MI, or stroke at 6 months.  ***Secondary*:** First occurrence of the composite of death, MI, or refractory ischaemia and the composite of death, MI, stroke, refractory ischaemia, or repeat intervention at 6 months. Other outcomes included each of the individual components analysed separately. |
| **Ethical Review** | The study was approved by the ethics committees of the participating hospitals. |
| **Overall bias assessment** | Low risk |
| ICA: invasive coronary angiography; Tn: troponin; h: hours; MI: myocardial infarction;  ECG: electrocardiogram; CKMB: creatin kinase muscle/brain; UA: unstable angina; NSTEMI: non-ST elevation myocardial infarction; ULN: upper limit of the normal; LMWH: low molecular weight heparin; ACS: acute coronary syndrome. | |

**Supplementary table 6. Key information trial**

**Kofoed et al., 2018 (VERDICT)**

| **Principal Investigator** | K F Kofoed |
| --- | --- |
| **Country** | Denmark |
| **Study Period** | November 2010 - June 2016 |
| **Study Design** | ***Very early:*** patients were transferred immediately from the referring hospital to the invasive center for ICA and possible revascularization, except during the night, when transfer was postponed to the early morning for logistical reasons.  ***Delayed:*** patients were transferred within 48 to 72 hours to the invasive centre. |
| **Inclusion Criteria** | At least 18 years of age, clinical suspicion of ACS, and ≥1 of the following high-risk criteria: (1) ECG changes indicating new ischaemia (new ST segment depression, horizontal or down sloping ≥0.05 mV in 2 consecutive leads, or T-wave inversion >0.01 mV in 2 leads with prominent R wave or R/S ratio >1); and (2) an increase in coronary markers of ischaemia (Tn). |
| **Exclusion Criteria** | Pregnancy, patient inability to understand trial information, indication for acute ICA (very high-risk NSTE-ACS, including ongoing ischaemia despite IV nitroglycerin infusion, haemodynamic or electric instability, acute HF, mechanical complication, or cardiac arrest), expected survival <1 year, and known intolerance to platelet inhibitors, heparin, or x-ray contrast, which could not be remedied medically. |
| **Follow up visits** | Information not available |
| **Outcomes** | ***Primary*:** Combination of all-cause death, nonfatal recurrent MI, hospital admission for refractory myocardial ischaemia, or hospital admission for HF.  ***Secondary*:** Invasive procedure complications during index hospitalisation (death, bleeding by the BARC criteria, procedure-related nonfatal acute MI, stroke, or TIA) in addition to the occurrence of each of the following events at any time after randomization: death, nonfatal acute MI, admission for refractory myocardial ischaemia, repeat coronary revascularization, or hospital admission for HF. |
| **Ethical Review** | The study was approved by the ethics committees of the participating hospitals. |
| **Overall bias assessment** | Low risk |
| ACS: acute coronary syndrome; Tn: troponin; ICA: invasive coronary angiography; NSTE-ACS: non-ST elevation acute coronary syndrome; IV: intravenous; MI: myocardial infarction; HF: heart failure; TIA: transient ischaemic attack. | |

**Supplementary table 7. Key information trial**

**Thiele et al., 2012 (LIPSIA-NSTEMI)**

| **Principal Investigator** | H Thiele |
| --- | --- |
| **Country** | Germany |
| **Study Period** | May 2006 – November 2009 |
| **Study Design** | Randomised, multicentre trial.  ***Immediate invasive:*** patients were scheduled to undergo angiography as soon as possible within the first 2 h after randomization.  ***Early invasive:*** patients were scheduled to undergo angiography on the next working day which was defined by a time window of 10–48 h after randomization.  ***Selectively invasive:*** patients were initially treated medically. |
| **Inclusion Criteria** | Patients with NSTEMI with ischaemic symptoms that were increasing or occurred at rest, with the last episode 24 h before randomization plus elevated Tn T level ≥ 0.1ng/mL. |
| **Exclusion Criteria** | Age <18 and >90 years, refractory ischaemia, haemodynamic instability, overt congestive HF, major arrhythmias requiring immediate catheterization, OAC, contraindications to heparin, aspirin, clopidogrel, and GPIIb/IIIa inhibitors, comorbidity with life expectancy <6 months, and alternate possibilities of Tn elevation. |
| **Follow up visits** | 6 months |
| **Outcomes** | ***Primary*:** CK-MB activity during index hospitalisation for each patient. In addition, the infarct size was estimated based on the AUC of CK-MB release.  ***Secondary*:** Composite of (i) death and non-fatal MI; (ii) death, non-fatal MI, and refractory ischaemia; (iii) death, non-fatal MI, refractory ischaemia, and rehospitalisation for UA within 6 months. |
| **Ethical Review** | The study was approved by the ethics committees of the participating hospitals. |
| **Overall bias assessment** | Low risk |
| Tn: troponin; NSTEMI: non-ST elevation myocardial infarction; MI: myocardial infarction; CK-MB: creatin kinase muscle/brain; UA: unstable angina; OAC: oral anticoagulation. GPIIb/IIIa: glycoprotein GPIIb/IIIa; AUC: area under the curve. | |

**Supplementary table 8. Key information trial**

**Milosevic et al., 2016 (RIDDLE-NSTEMI)**

| **Principal Investigator** | G Stankovic |
| --- | --- |
| **Country** | Serbia |
| **Study Period** | September 2009 - February 2013 |
| **Study Design** | Randomized, parallel-group, open-label, single-centre trial.  ***Early:*** Patients were transferred for angiography as soon as possible but no later than 2 h after randomization.  ***Delayed:*** Patients underwent invasive intervention within 72 h of randomization, except for patients in whom chest pain developed, who had recurrent ischaemia, and/or who became clinically unstable while waiting to undergo the intervention. |
| **Inclusion Criteria** | Patients with an episode of chest pain occurring no more than 24 h before admission and if the following 2 findings were present on admission to the hospital: 1) elevation of cardiac Tn I greater than the ULN and 2) new ST-segment depression at least 1 mV and/or T-wave inversion in ≥ 2 contiguous leads. |
| **Exclusion Criteria** | Persistent ST-segment elevation, posterior MI, haemodynamic instability, cardiogenic shock, life-threatening ventricular arrhythmias, and/or refractory angina on admission, active bleeding, any contraindication for the use of DAPT, and the presence of comorbidities with a life expectancy <6 months. |
| **Follow up visits** | 30 days and 1 year |
| **Outcomes** | ***Primary*:** Composite of death from any cause or new MI at 30-day follow-up.  ***Secondary*:** A combined incidence of death, new MI, and/or recurrent ischaemia at 30 days and 1 year, as well as death or new MI at 1 year. Rates of individual components of the primary and secondary endpoints and major bleedings at 30 days and 1 year. |
| **Ethical Review** | The study was approved by the ethics committees of the participating hospitals. |
| **Overall bias assessment** | Low risk |
| Tn: troponin; ULN: upper limit of normal; DAPT: dual antiplatelet therapy; MI: myocardial infarction. | |

**Supplementary table 9. Univariable and multivariable Cox regression analysis using fixed effect for the comparison between female and male sex in the early and delayed invasive strategy groups**

| **Early strategy**  **N= 3407** | | |
| --- | --- | --- |
|  | **HR (95%CI)** | **P-value** |
| **Primary endpoint** | | |
| Female Sex |  |  |
| Univariable unadjusted model | 1.10 (0.85-1.44) | 0.464 |
| Multivariable adjusted model^*^ | 0.97 (0.74-1.26) | 0.795 |
| **All-cause death** | | |
| Female Sex |  |  |
| Univariable unadjusted model | 1.18 (0.84-1.67) | 0.345 |
| Multivariable adjusted model^*^ | 1.05 (0.74-1.50) | 0.770 |
| **Myocardial infarction** | | |
| Female Sex |  |  |
| Univariable unadjusted model | 1.17 (0.81-1.68) | 0.410 |
| Multivariable adjusted model^*^ | 1.01 (0.70-1.46) | 0.958 |
| **Recurrent ischaemia** | | |
| Female Sex |  |  |
| Univariable unadjusted model | 0.79 (0.52-1.19) | 0.262 |
| Multivariable adjusted model^*^ | 0.74 (0.48-1.15) | 0.186 |
| **Stroke** | | |
| Female Sex |  |  |
| Univariable unadjusted model | 0.58 (0.22-1.58) | 0.290 |
| Multivariable adjusted model^*^ | 0.53 (0.19-1.46) | 0.219 |
| **Major bleedings** |  |  |
| Female Sex |  |  |
| Univariable unadjusted model | 0.86 (0.52-1.41) | 0.542 |
| Multivariable adjusted model^*^ | 0.79 (0.47-1.33) | 0.372 |
| **Delayed strategy**  **N= 3247** | | |
| **Primary endpoint** | | |
| Female Sex |  |  |
| Univariable unadjusted model | 1.12 (0.88-1.43) | 0.355 |
| Multivariable adjusted model^*^ | 0.93 (0.72-1.20) | 0.570 |
| **All-cause death** | | |
| Female Sex |  |  |
| Univariable unadjusted model | 1.37 (0.99-1.91) | 0.059 |
| Multivariable adjusted model^*^ | 1.09 (0.78-1.54) | 0.609 |
| **Myocardial infarction** |  |  |
| Female Sex |  |  |
| Univariable unadjusted model | 0.97 (0.70-1.35) | 0.863 |
| Multivariable adjusted model^*^ | 0.82 (0.58-1.16) | 0.261 |
| **Recurrent ischaemia** | | |
| Female Sex |  |  |
| Univariable unadjusted model | 1.44 (1.00-2.07) | 0.049 |
| Multivariable adjusted model^*^ | 1.34 (0.91-1.97) | 0.144 |
| **Stroke** | | |
| Female Sex |  |  |
| Univariable unadjusted model | 1.45 (0.68-3.07) | 0.330 |
| Multivariable adjusted model^*^ | 1.27 (0.58-2.79) | 0.546 |
| **Major bleedings** |  |  |
| Female Sex |  |  |
| Univariable unadjusted model | 1.81 (1.18-2.77) | 0.006 |
| Multivariable adjusted model^*^ | 1.30 (0.81-2.09) | 0.279 |
| * Multivariable adjusted model for age, diabetes mellitus, biomarkers status and GRACE score. CI: confidence interval; HR: hazard ratio; N: number. | | |

**Supplementary table 10. 6-month clinical outcomes stratified by sex in early and delayed invasive strategy cohorts of patients with GRACE score ≤140 using random effect Cox model**

| **Low-risk patients**  **GRACE ≤140 (N=3833)** | | | | | | | | | |
| --- | --- | --- | --- | --- | --- | --- | --- | --- | --- |
|  | **Early strategy**  **(N=1978)** | | | | **Delayed strategy**  **(N=1855)** | | | |  |
| **Endpoints** | **Females**  **(N=638)** | **Males**  **(N=1340)** | **HR**  **(95% CI)** | **P-value** | **Females**  **(N=591)** | **Males**  **(N=1264)** | **HR**  **(95% CI)** | **P-value** | **P-interaction** |
| Primary endpoint | 39 (6.1) | 58 (4.3) | 1.42  (0.94-2.12) | 0.094 | 27 (4.6) | 60 (4.7) | 0.98  (0.62-1.54) | 0.920 | 0.220 |
| All-cause death | 18 (2.8) | 23 (1.7) | 1.66  (0.90-3.08) | 0.110 | 13 (2.2) | 15 (1.2) | 1.90  (0.90-3.99) | 0.090 | 0.780 |
| Myocardial infarction | 24 (3.8) | 37 (2.8) | 1.35  (0.81-2.26) | 0.250 | 17 (2.9) | 49 (3.9) | 0.74  (0.43-1.29) | 0.290 | 0.110 |
| Recurrent ischaemia | 17 (2.7) | 29 (2.2) | 1.31  (0.72-2.38) | 0.380 | 19 (3.2) | 32 (2.5) | 1.30  (0.74-2.30) | 0.360 | 0.950 |
| Stroke | 2 (0.3) | 11 (0.8) | 0.38  (0.08-1.71) | 0.210 | 4 (0.7) | 7 (0.6) | 1.21  (0.35-4.13) | 0.760 | 0.240 |
| Major bleedings | 12 (1.9) | 29 (2.2) | 0.85  (0.43-1.67) | 0.640 | 19 (3.2) | 18 (1.4) | 2.34  (1.23-4.46) | 0.010 | 0.033 |
| CI: confidence interval; HR: hazard ratio; N: number. | | | | | | | | | |

**Supplementary table 11. 6-month clinical outcomes stratified by sex in early and delayed invasive strategy cohorts of patients with GRACE score >140 using random effect Cox model**

| **High-risk patients**  **GRACE >140 (N=2539)** | | | | | | | | | |
| --- | --- | --- | --- | --- | --- | --- | --- | --- | --- |
|  | **Early strategy**  **(N=1296)** | | | | **Delayed strategy**  **(N=1243)** | | | |  |
| **Endpoints** | **Females** | **Males** | **HR**  **(95% CI)** | **P-value** | **Females**  **(N=480)** | **Males**  **(N=763)** | **HR**  **(95% CI)** | **P-value** | **P-interaction** |
| Primary endpoint | 47 (10.1) | 93 (11.2) | 0.88  (0.62-1.25) | 0.470 | 74 (15.4) | 108 (14.2) | 1.09  (0.81-1.47) | 0.560 | 0.310 |
| All-cause death | 33 (7.1) | 58 (7.0) | 0.98  (0.64-1.50) | 0.920 | 47 (9.8) | 63 (8.3) | 1.18  (0.81-1.73) | 0.380 | 0.490 |
| Myocardial infarction | 22 (4.7) | 41 (4.9) | 0.95  (0.57-1.60) | 0.860 | 34 (7.1) | 54 (7.1) | 1.00  (0.65-1.54) | 1.000 | 0.820 |
| Recurrent ischaemia | 12 (2.6) | 38 (4.6) | 0.56  (0.29-1.07) | 0.080 | 28 (5.8) | 30 (3.9) | 1.69  (1.01-2.83) | 0.048 | 0.012 |
| Stroke | 3 (0.6) | 6 (0.7) | 0.82  (0.21-3.28) | 0.780 | 8 (1.7) | 7 (0.9) | 1.68  (0.61-4.65) | 0.320 | 0.400 |
| Major bleedings | 9 (1.9) | 15 (1.8) | 0.95  (0.41-2.16) | 0.900 | 14 (2.9) | 20 (2.6) | 1.03  (0.52-2.04) | 0.930 | 0.900 |
| CI: confidence interval; HR: hazard ratio; N: number. | | | | | | | | | |

**Supplementary table 12. 6-month clinical outcomes stratified by sex in early and delayed invasive strategy cohorts of patients with GRACE score ≤140 using fixed effect Cox model**

| **GRACE ≤140** | | | | | |
| --- | --- | --- | --- | --- | --- |
|  | **Early strategy** | | **Delayed strategy** | |  |
| **Endpoints** | **HR**  **(95% CI)** | **P-value** | **HR**  **(95% CI)** | **P-value** | **P-interaction** |
| Primary endpoint | 1.44  (0.96-2.16) | 0.079 | 0.96  (0.61-1.51) | 0.865 | 0.193 |
| All-cause death | 1.66  (0.90-3.08) | 0.106 | 1.87  (0.89-3.92) | 0.100 | 0.813 |
| Myocardial infarction | 1.38  (0.82-2.30) | 0.223 | 0.74  (0.43-1.29) | 0.291 | 0.109 |
| Recurrent ischaemia | 1.24  (0.68-2.26) | 0.478 | 1.29  (0.73-2.28) | 0.380 | 0.924 |
| Stroke | 0.38  (0.09-1.73) | 0.213 | 1.23  (0.36-4.19) | 0.744 | 0.241 |
| Major bleedings | 0.87  (0.45-1.71) | 0.691 | 2.28  (1.20-4.34) | 0.012 | 0.044 |
| CI: confidence interval; HR: hazard ratio. | | | | | |

**Supplementary Table 13. 6-month clinical outcomes stratified by sex in early and delayed invasive strategy cohorts of patients with GRACE score >140 using fixed effect Cox model**

| **GRACE >140** | | | | | |
| --- | --- | --- | --- | --- | --- |
|  | **Early strategy** | | **Delayed strategy** | |  |
| **Endpoints** | **HR**  **(95% CI)** | **P-value** | **HR**  **(95% CI)** | **P-value** | **P-interaction** |
| Primary endpoint | 0.91  (0.64-1.29) | 0.587 | 1.12  (0.84-1.51) | 0.442 | 0.363 |
| All-cause death | 1.00  (0.65-1.54) | 0.989 | 1.20  (0.82-1.76) | 0.339 | 0.535 |
| Myocardial infarction | 0.95  (0.57-1.60) | 0.853 | 1.01  (0.66-1.55) | 0.960 | 0.864 |
| Recurrent ischaemia | 0.55  (0.29-1.05) | 0.071 | 1.51  (0.90-2.53) | 0.118 | 0.017 |
| Stroke | 0.88  (0.22-3.53) | 0.861 | 1.85  (0.67-5.09) | 0.236 | 0.398 |
| Major bleedings | 1.06  (0.47-2.43) | 0.887 | 1.13  (0.57-2.24) | 0.724 | 0.909 |
| CI: confidence interval; HR: hazard ratio. | | | | | |

**Supplementary Table 14. Univariable Cox regression analysis using random effect for the comparison between female and male sex among high-risk patients undergoing invasive strategy before or after 24 hours from randomisation.** *High-risk* was defined according to the current guidelines criteria considering a GRACE score >140 and/or the presence of transient ST-segment elevation at baseline ECG.

| **High-risk patients**  **(n=6247)** | | | | | | | | | |
| --- | --- | --- | --- | --- | --- | --- | --- | --- | --- |
|  | **Invasive strategy**  **performed before 24h**  **(n=3556)** | | | | **Invasive strategy**  **performed after 24h**  **(n=2691)** | | | |  |
| **Endpoints** | **Females**  **N=1211** | **Males**  **N=2345** | **HR^*^**  **(95% CI)** | **P-value** | **Females**  **N=922** | **Males**  **N=1769** | **HR^*^**  **(95% CI)** | **P-value** | **P-interaction** |
| Primary  endpoint | 99  (8.18) | 172  (7.34) | 1.14  (0.89-1.47) | 0.290 | 72  (7.81) | 129  (7.29) | 1.08  (0.81-1.44) | 0.610 | 0.760 |
| All-cause  death | 58  (4.79) | 79  (3.37) | 1.45  (1.03-2.03) | 0.320 | 40  (4.34) | 63  (3.56) | 1.23  (0.82-1.82) | 0.320 | 0.530 |
| Myocardial infarction | 53  (4.38) | 102  (4.35) | 1.03  (0.74-1.44) | 0.850 | 40  (4.34) | 74  (4.18) | 1.04  (0.71-1.52) | 0.850 | 0.980 |
| Recurrent ischaemia | 42  (3.47) | 85  (3.63) | 1.05  (0.72-1.52) | 0.810 | 29  (3.15) | 46  (2.60) | 1.29  (0.81-2.06) | 0.280 | 0.520 |
| Stroke | 5  (0.41) | 18  (0.77) | 0.55  (0.20-1.47) | 0.230 | 10  (1.09) | 13  (0.74) | 1.48  (0.64-3.37) | 0.360 | 0.140 |
| Major  bleedings | 25  (2.06) | 53  (2.26) | 0.94  (0.58-1.51) | 0.800 | 36  (3.91) | 39  (2.21) | 1.83  (1.16-2.88) | 0.009 | 0.063 |
| * HR and 95% CI for the comparison between early and delayed strategy using random effect Cox model to adjust for within-study clustering. Univariable unadjusted model for treatment strategy.  CI: confidence interval; HR: hazard ratio; N: number. | | | | | | | | | |

**Supplementary figure 1. Studies selection flow chart**

2232 records identified

from literature source

IPD: 6654 patients

940 duplicates removed

1292 titles and abstracts screened

1274 excluded

18 studies reviewed

(full-text)

12 excluded:

4 not provided response

3 small trials with few female participants

1 enrolling UA only

1 randomised after angiography

1 unable to contact author

1 comparison to selective invasive treatment

1 pre-hospital randomisation

6 RCTs selected and provided IPD

N= 6656 patients

2 patients excluded

(lack of follow-up data)

**Supplementary figure 2. Median time (hours) between randomisation process and coronary angiography in the early and delayed invasive strategy arms of the six included trials.** Boxes correspond to interquartile ranges, the vertical lines to the median value.


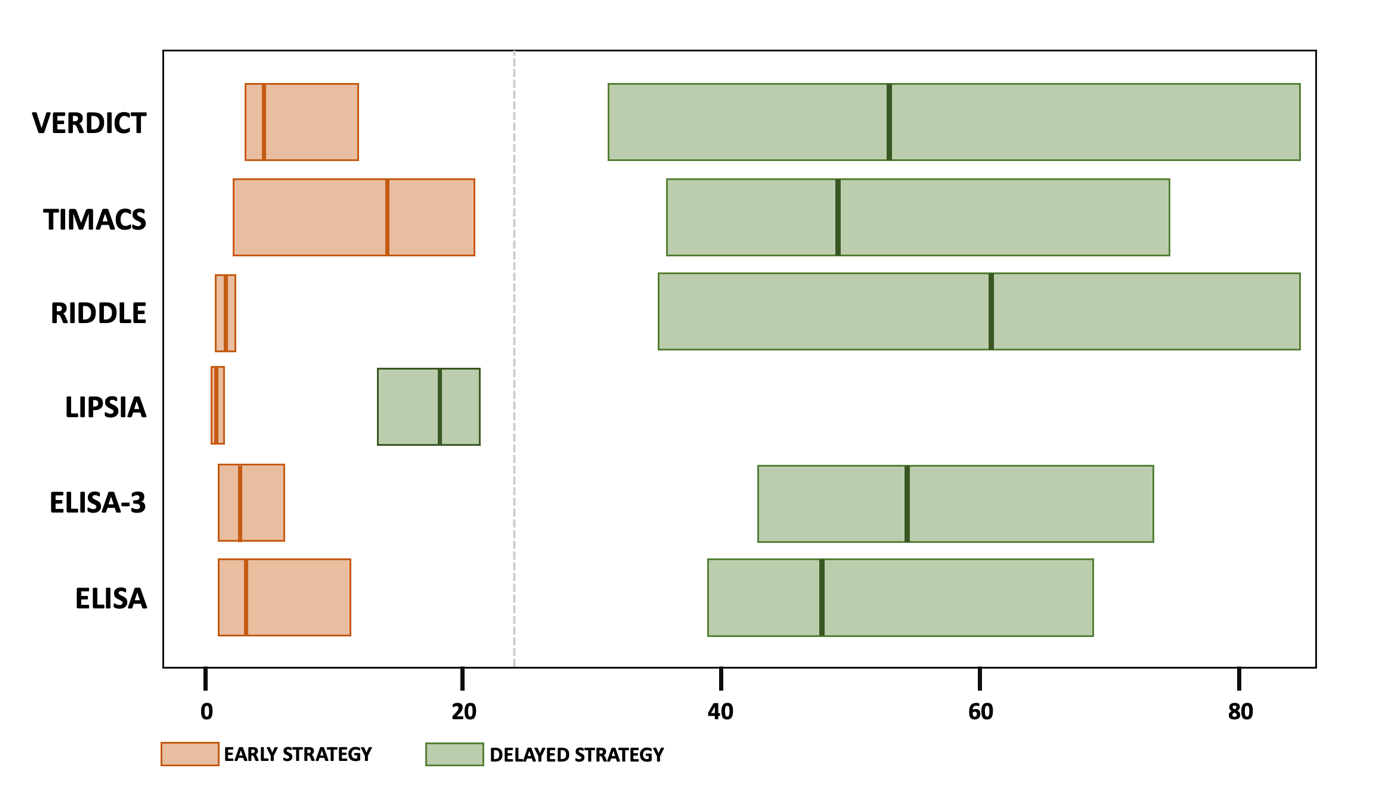


**Supplementary figure 3. Primary and secondary endpoints in patients undergoing early invasive strategy stratified by sex.** p_LR_= p-log rank.

**
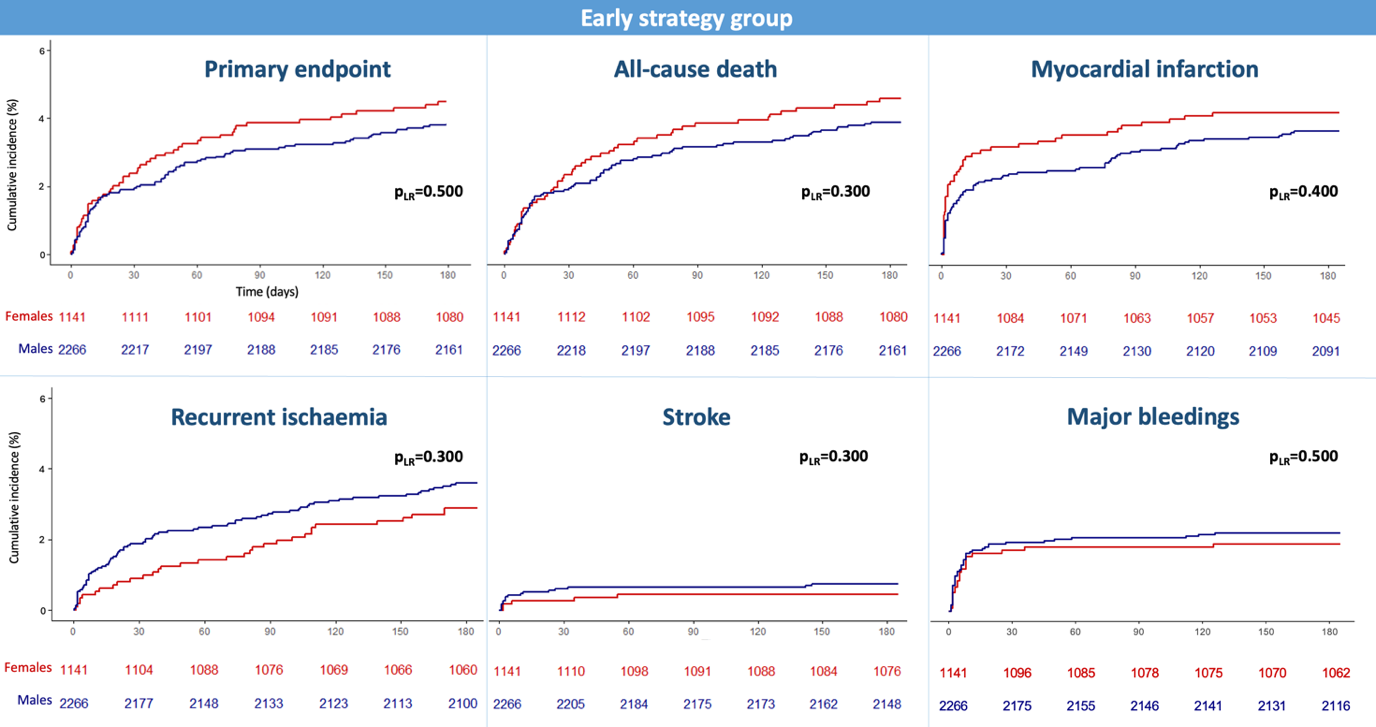
**

**Supplementary figure 4. Primary endpoint, all-cause death, myocardial infarction and stroke in patients undergoing delayed invasive strategy stratified by sex.** p_LR_= p-log rank.


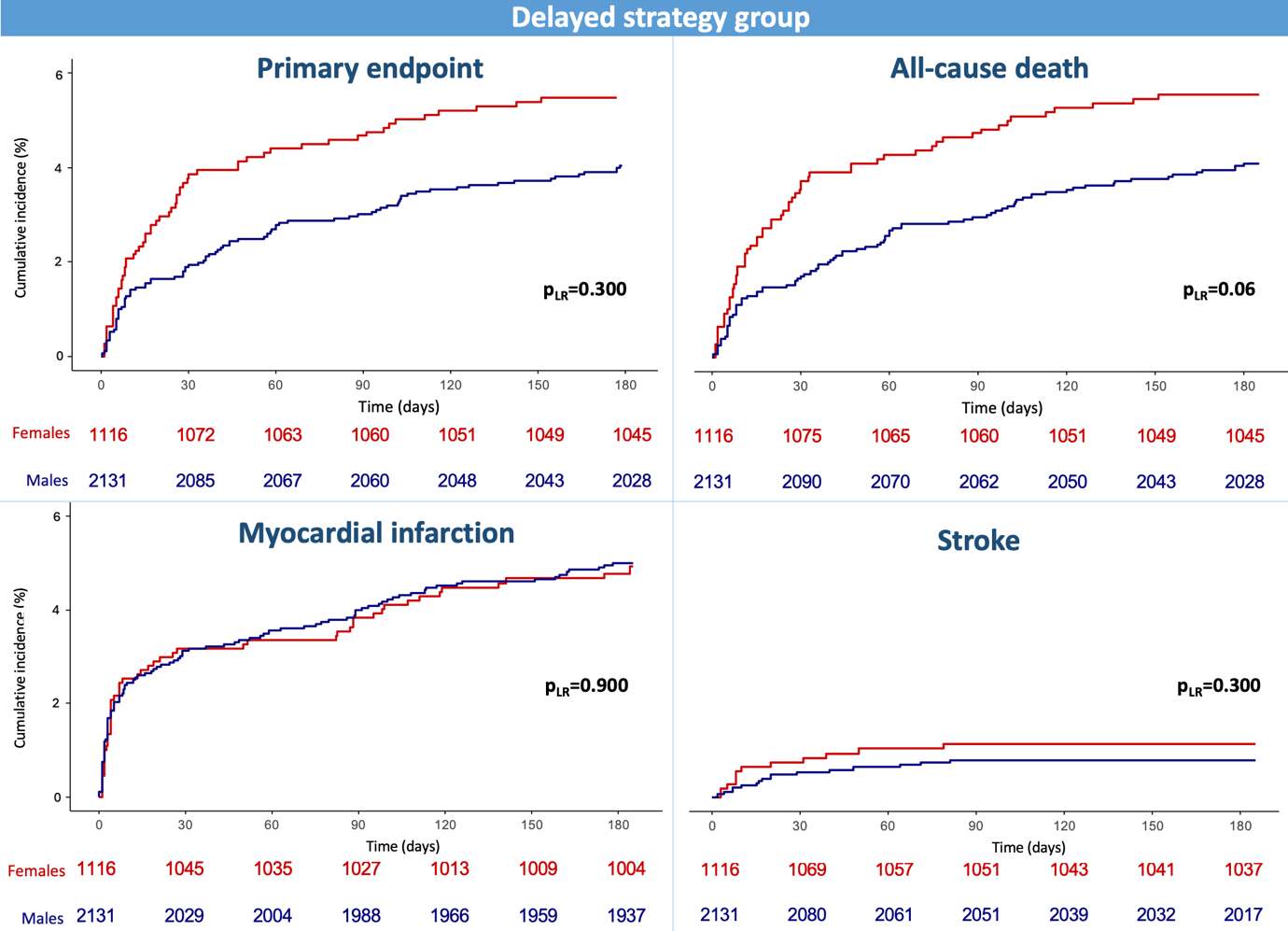


**Supplementary figure 5. Two-stage meta-analysis including all the six randomised controlled trials for the endpoint myocardial infarction.**

**
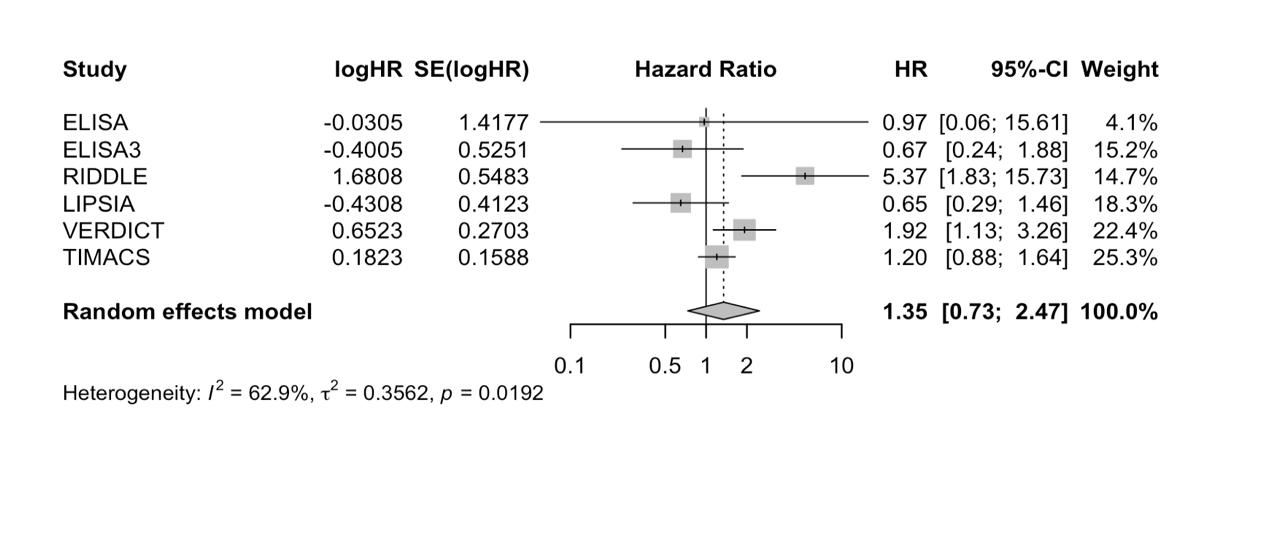
**

**Supplementary figure 6. Scatter plot displaying the most influent studies impacting on between-trial heterogeneity for myocardial infarction.**

**
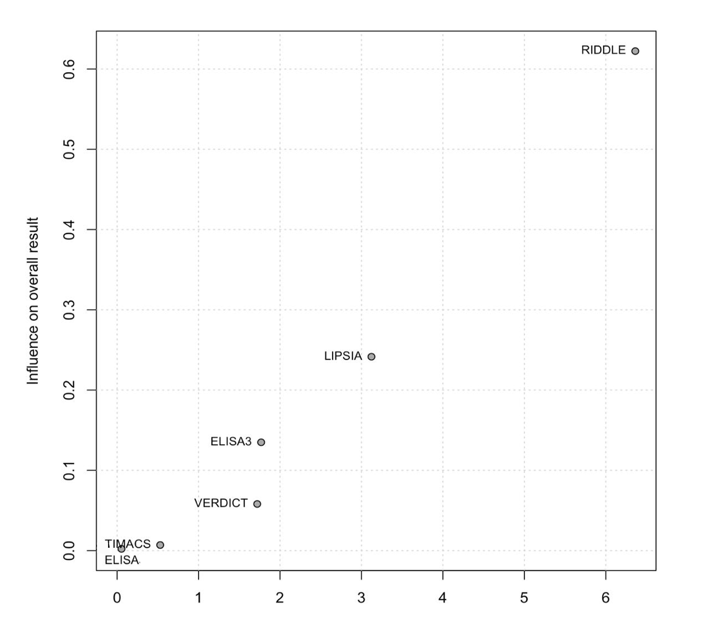
**

**Supplementary figure 7. Two-stage meta-analysis for the endpoint myocardial infarction after exclusion of the three RCTs (ELISA-3, RIDDLE and LIPSIA) that most influenced the between-trial heterogeneity.**

**
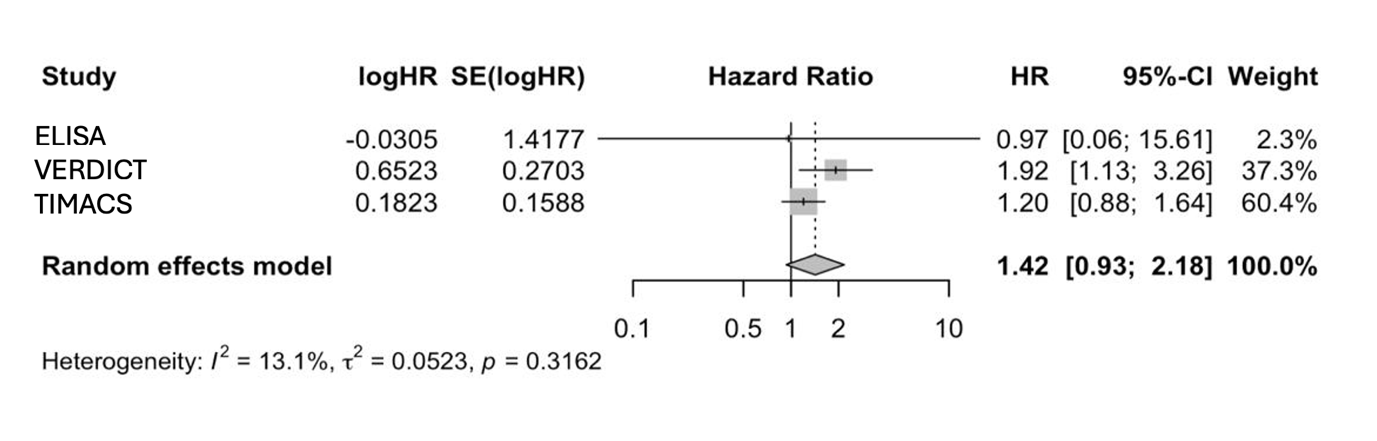
**

**Supplementary figure 8. Two-stage meta-analysis including all the six randomised controlled trials for the endpoint recurrent ischaemia.**

**
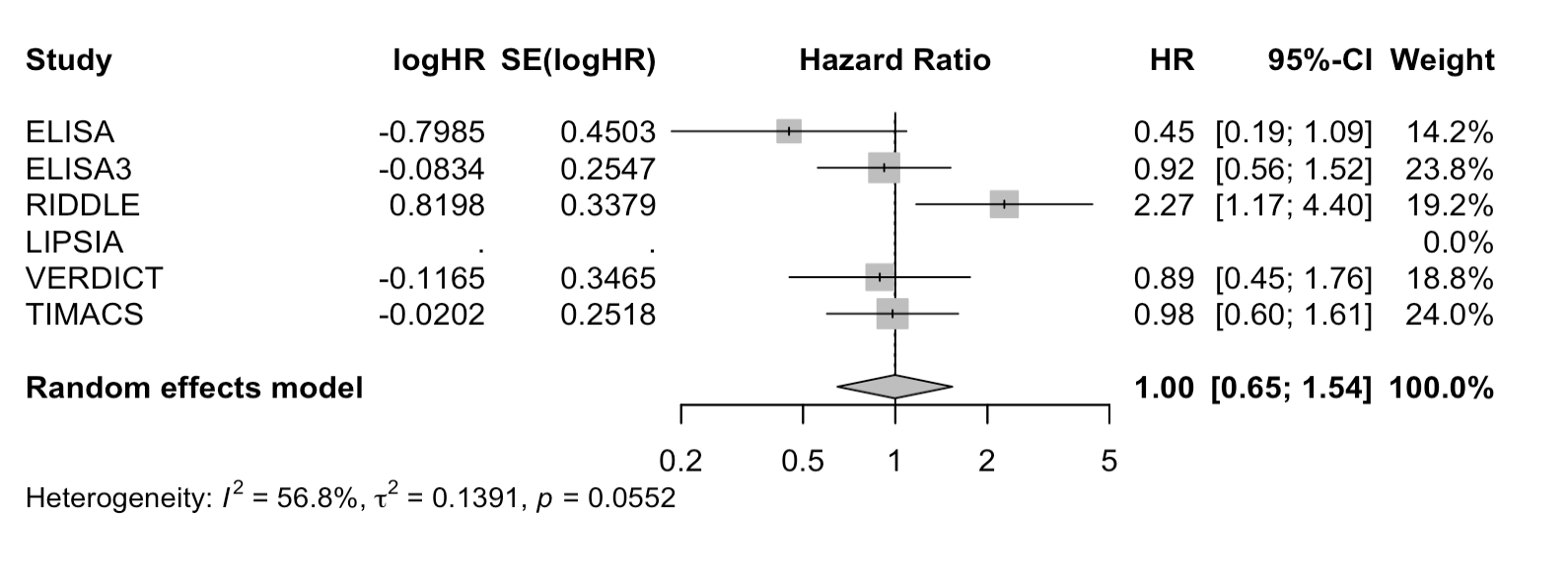
**

**Supplementary figure 9. Scatter plot displaying the most influent studies impacting on between-trial heterogeneity for the endpoint recurrent ischaemia.**

# **
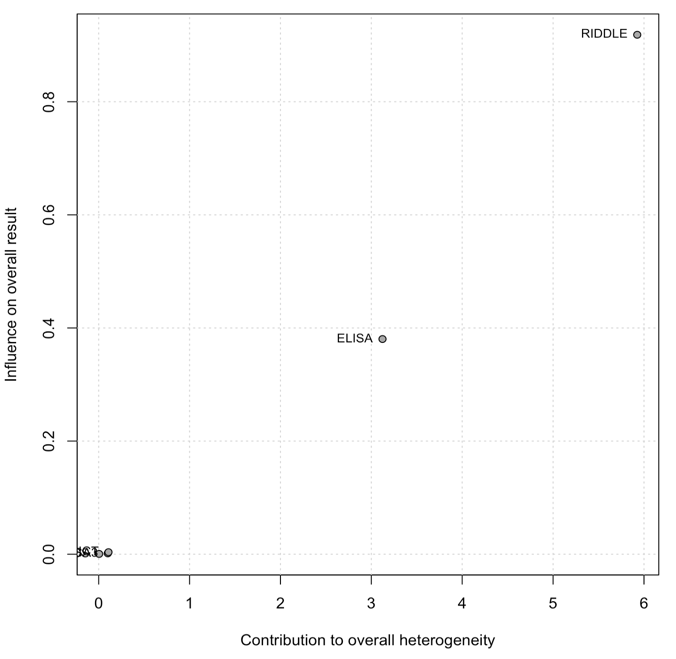
**

**Supplementary figure 10. Two-stage meta-analysis for the endpoint MI after exclusion of the two RCTs (RIDDLE and ELISA) that most influenced the between-trial heterogeneity.**

**
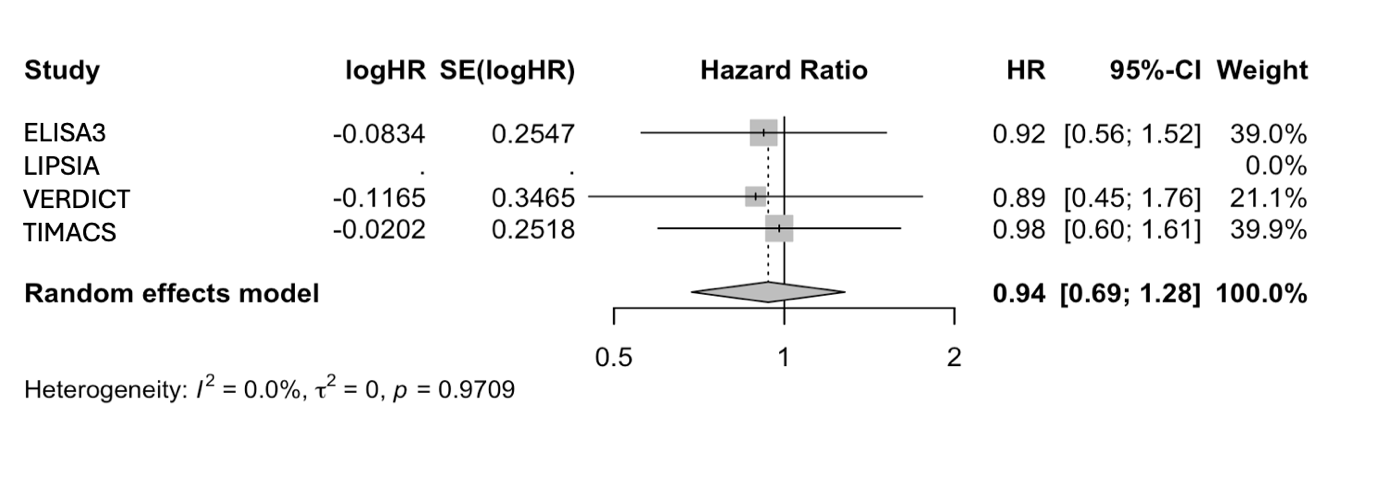
**

## **Supplementary figure 11. Publication bias for the endpoints.**


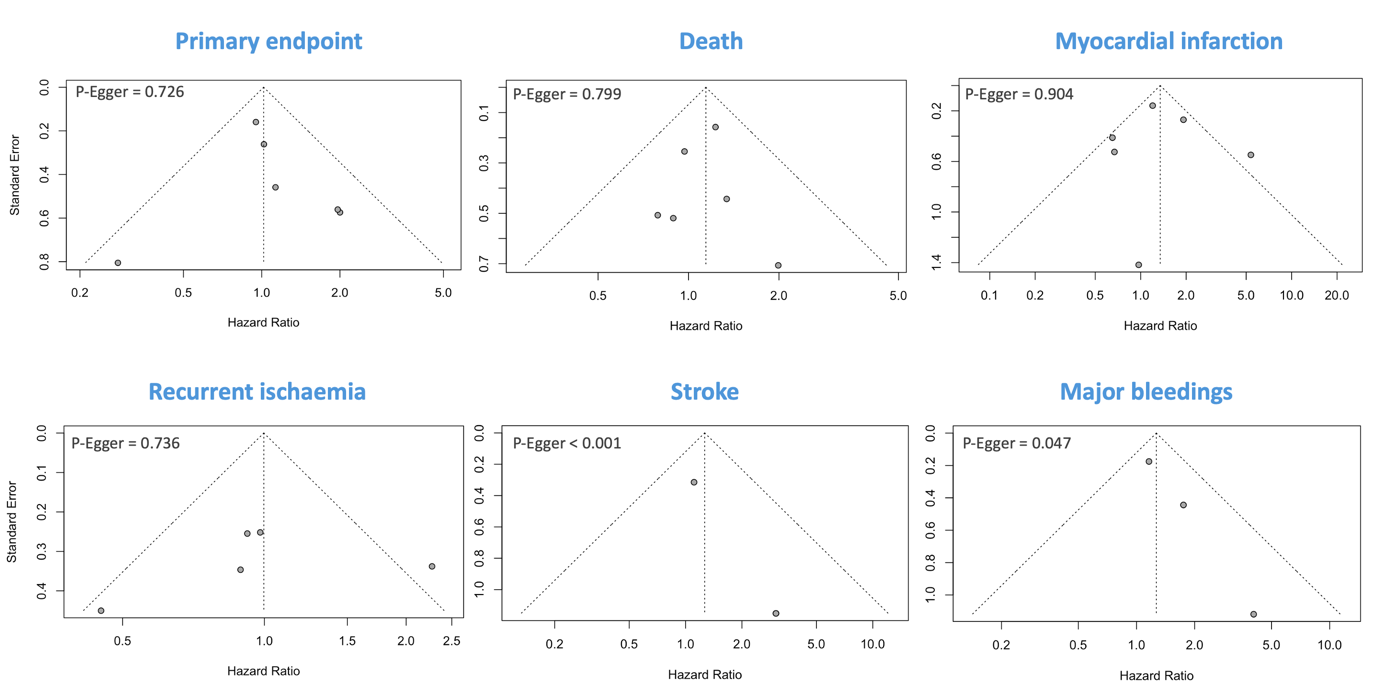


# The plot displays funnel plots for each endpoint, with the results of Egger’s test presented in the top-left corner for each endpoint.

# **Supplementary figure 12. Cochrane risk-of-bias tool for randomised trials version 2 (RoB 2). Green colour corresponds to low risk of bias, yellow to some concerns, and red to high risk of bias.**

|  | Randomisation process | Effect of assignment to intervention | Missing outcome data | Measurement of the outcome | Selection of the reported result | Overall risk of bias |
| --- | --- | --- | --- | --- | --- | --- |
| van ’t Hof 2003 (ELISA) |  |  |  |  |  |  |
| Mehta 2009 (TIMACS) |  |  |  |  |  |  |
| Thiele 2012  (LIPSIA-NSTEMI) |  |  |  |  |  |  |
| Badings 2013 (ELISA-3) |  |  |  |  |  |  |
| Milosevic 2016 (RIDDLE-NSTEMI) |  |  |  |  |  |  |
| Kofoed 2018 (VERDICT) |  |  |  |  |  |  |
